# Integrative Transcriptomic and Metabolomic Analysis Reveals That *Acanthopanax senticosus* Fruit Ameliorates Cisplatin-Induced Acute Kidney Injury by Suppressing the NF-κB/PI3K-AKT Pathway via UGT1A1 Regulation

**DOI:** 10.3390/ijms262211131

**Published:** 2025-11-18

**Authors:** Liu Han, Zebo Tang, Xiangyu Ma, Qiuyue Zhang, Yu Han, Qi Wang, Jinlong Liu, Xuefeng Bian, Liancong Gao, Mengran Xu, Xin Sun

**Affiliations:** 1 College of Pharmacy, Jilin Medical University, Jilin 132013, China; 2 College of Basic Medical Sciences, Jilin Medical University, Jilin 132013, China; 3 College of Pharmacy, Yanbian University, Yanji 133002, China; 4 Clinical Medical College, Jilin Medical University, Jilin 132013, China

**Keywords:** *Acanthopanax senticosus* fruit ethanol extract, acute kidney injury, cisplatin, multi-omics analysis

## Abstract

The chemical composition of the ethanol extract of *Acanthopanax senticosus* fruit (ASFEE) was systematically characterized using UPLC-MS/MS (Q Exactive Orbitrap), leading to the identification of 45 compounds. Through integrated network pharmacology and molecular docking analyses, the binding affinities between key bioactive constituents—such as eleutheroside E (EE) and quercetin—and core therapeutic targets were predicted and validated. A total of 125 overlapping targets were identified between ASFEE and acute kidney injury (AKI), with significant enrichment observed in critical signaling pathways, including NF-κB, IL-17, and PI3K-Akt. To evaluate the protective effects of ASFEE, both in vitro (HK-2 cells) and in vivo (murine) models of cisplatin (DDP)-induced AKI were employed. Parameters assessed included cell viability, apoptosis, reactive oxygen species (ROS) production, activation of the NF-κB signaling pathway, kidney function, histopathological alterations, and levels of inflammatory cytokines. ASFEE treatment markedly enhanced HK-2 cell viability and reduced cellular apoptosis and ROS generation. In the murine model, DDP administration resulted in significantly elevated serum creatinine (Scr) and blood urea nitrogen (BUN) levels. Both low- and high-dose ASFEE treatments significantly attenuated these increases, improved overall kidney function, and alleviated kidney tubular damage. Furthermore, ASFEE reduced serum levels of pro-inflammatory cytokines, including IL-1β, IL-6, and TNF-α. Multi-omics integration analysis enabled the identification of differentially expressed genes and metabolites. ASFEE was found to reverse 4689 DDP-induced gene expression changes and 323 metabolic disturbances, with the uridine diphosphate glucuronosyltransferase (UGT)-mediated ascorbic acid metabolism pathway emerging as the central regulatory axis. Key candidate genes and proteins were further validated via real-time quantitative polymerase chain reaction (RT-qPCR) and Western blotting. DDP significantly upregulated the expression of inflammatory markers and associated signaling molecules in kidney tissues, while concurrently downregulating UGT family genes and the UGT1A1 protein involved in uronic acid metabolism. Notably, ASFEE treatment effectively counteracted these alterations, confirming its role in enhancing UGT1A1-mediated metabolic processes and suppressing the NF-κB/PI3K-Akt/IL-17 signaling cascade. These mechanisms contribute to improved antioxidant capacity, mitigation of inflammatory responses, and restoration of metabolic homeostasis, thereby conferring protection against DDP-induced AKI. ASFEE exerts a protective effect on AKI caused by DDP by enhancing antioxidant capacity, inhibiting inflammation and restoring metabolic homeostasis, providing an experimental basis for its subsequent development and application.

## 1. Introduction

Acute kidney injury (AKI) is characterized by rapid deterioration of kidney function, with a high incidence and mortality rate. It is difficult to treat clinically and has a dangerous prognosis. Factors such as surgery, infection, hypovolemic shock, and sepsis can all lead to the occurrence of AKI. Annually, AKI is responsible for an estimated 2 million deaths around the world, with medical expenses exceeding 13 billion US dollars [1]. There are 2.9 million AKI patients in China each year, and the prevalence rate is increasing year by year [2]. The pathogenesis of AKI is complex. Currently, it is believed that the occurrence of AKI involves multiple regulatory pathways of cell death and may be related to factors such as oxidative stress, inflammatory response, and mitochondrial dysfunction [3]. The application of nephrotoxic drugs is a common cause of AKI. Cisplatin (DDP) is one of the most widely used and successful anti-cancer drugs in clinical practice today. However, its clinical application is severely limited due to its easy occurrence of toxic side effects such as kidney, cardiac and gastrointestinal toxicity [4]. The nephrotoxicity of DDP, a common chemotherapeutic drug, involves a cascade of events. It induces acute kidney injury by promoting oxidative stress through reactive oxygen species (ROS) generation, instigating a robust inflammatory response, and causing direct DNA damage. These mechanisms collectively lead to cellular dysfunction, apoptosis, and programmed necrosis in renal tubular cells [5,6,7]. At present, apart from blood purification, there are few other effective treatment methods for AKI. Untreated AKI will further aggravate kidney damage and is closely related to other adverse prognostic events. Therefore, it is imperative to identify novel therapeutic targets and agents for AKI management.

*Acanthopanax senticosus* (AS) is a plant of the Araliaceae family. Its roots and rhizomes are used as medicine. Recent pharmacological studies have demonstrated the kidney protective effects of AS in rat models of kidney ischemia–reperfusion injury. Specifically, AS treatment was found to enhance the activity of superoxide dismutase (SOD) in kidney tissues, thereby strengthening antioxidant capacity and reducing oxidative damage. Furthermore, AS administration significantly suppressed tumor necrosis factor-α (TNF-α) levels, alleviated inflammatory injury, decreased serum creatinine (Scr) concentrations, and improved pathological changes in kidney tissues. These combined effects resulted in substantial improvement of kidney function [8,9]. Although the roots and stems of *Acanthopanax senticosus* have been widely used, its fruit, as a potential resource, has received less attention. Recent studies have found that the fruits of *Acanthopanax senticosus* are also rich in various active components, such as flavonoids and eleutherosides [10], and have shown good antioxidant and anti-inflammatory potential [11]. Therefore, this study aims to systematically evaluate the kidney protective effect of ethanol extract of *Acanthopanax senticosus* fruit (ASFEE).

In recent years, multi-omics approaches that integrate transcriptomics, metabolomics and other omics technologies have become powerful tools for systematically studying the complex molecular networks behind physiological and pathological processes. By comprehensively analyzing the changes in gene expression and metabolite profiles, multi-omics can identify key molecular targets, signaling pathways, and metabolic disorders related to disease development and treatment responses. This holistic approach is particularly valuable for clarifying the complex mechanisms of action of natural products, as they typically exert their effects through multiple targets and pathways [12].

Against this backdrop, this study aims to explore the protective effect of ASFEE on AKI by using a multi-omics strategy. By integrating network pharmacology, transcriptomics and metabolomics analyses, we attempt to reveal the global molecular changes induced by DDP and the regulatory role of ASFEE. This comprehensive study is expected to identify the key biological processes, key molecules and interrelated pathways involved in ASFEE mediated kidney protection, providing new insights into the therapeutic potential of ASFEE and laying the foundation for the development of effective AKI intervention measures.

## 2. Results

### 2.1. Analysis of Chemical Constituents in ASFEE by UPLC-MS/MS 

**Figure 1:**
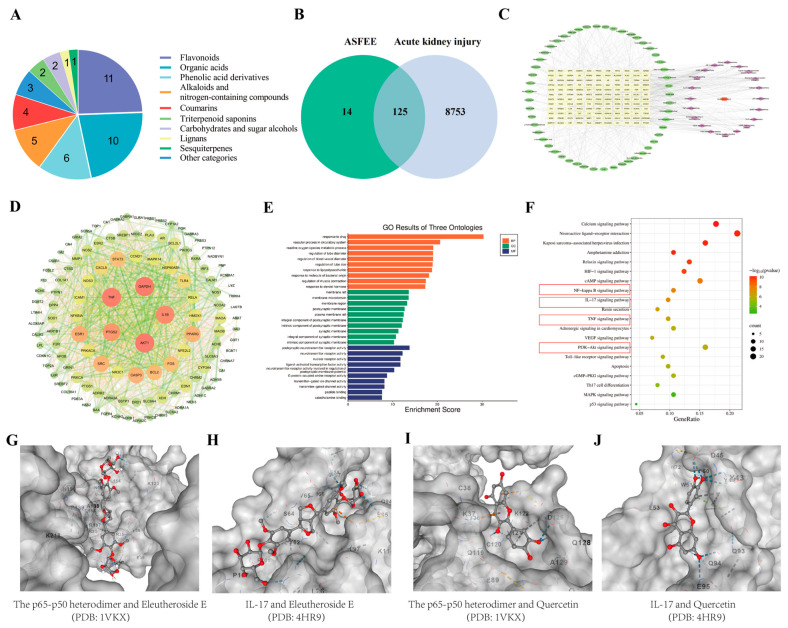
Chemical composition detection and network pharmacology of ASFEE. (**A**) Structural classification of compounds contained in ASFEE using (UPLC-MS/MS) (Q Exactive Orbitrap); (**B**) Venn diagram of overlapping targets between ASFEE and AKI; (**C**) Drug-disease-target network visualization; (**D**) PPI network diagram; (**E**) Bar chart representing the results of GO Enrichment Analysis; (**F**) Bubble plots of KEGG; (**G**) Molecular docking results of the p65-p50 heterodimer and Eleutheroside E; (**H**) Molecular docking results of IL-17 and Eleutheroside E; (**I**) Molecular docking results of the p65-p50 heterodimer and Quercetin; (**J**) Molecular docking results of IL-17 and Quercetin. Red represents oxygen atoms, gray represents carbon atoms, and the blue dotted line represents hydrogen bonds in (**G**–**J**).

The ASFEE was characterized using UPLC-MS/MS (Q Exactive Orbitrap) (Thermo Fisher Scientific, Waltham, MA, USA). Representative total ion chromatograms in both positive and negative ion modes are shown in Figures S1 and S2. By combining precise quality measurements, MS/MS fragment patterns, comparisons with reference standards, published literature data, and HMDB, METLIN, and mzCloud databases, a total of 45 components were identified or preliminarily identified from the ethanol extract. These components are classified into 9 major categories (Figure 1A and Table S2): Flavonoids (11 compounds), Organic acids (10 compounds), Phenolic acid derivatives (6 compounds) Alkaloids and nitrogen-containing compounds (5 compounds), Coumarins (4 compounds), Triterpenoid saponins (2 compounds) Carbohydrates and sugar alcohols (2 compounds), Lignans (1 compound), Sesquiterpenes (1 compound) Other categories (3 compounds).

### 2.2. Analysis of Network Pharmacology

#### 2.2.1. Drug Targets of ASFEE and Disease Targets of AKI

In the TCMSP database, 139 targets of ASFEE were obtained and transformed through the UniProt database. In the Gene Cards and OMIM databases, 8878 AKI disease targets were collected. A total of 125 cross-targets were identified through the Venny method, which are potential targets for ASFEE in the treatment of AKI (Figure 1B).

The active ingredients and their corresponding targets were imported into STRING and Cytoscape 3.8.2 software to construct and analyze the protein–protein interaction (PPI) network between ASFEE and AKI cross-targets (Figure 1C). Based on the comprehensive ranking of degree centrality (DC), intermediate centrality (BC), and proximity centrality (CC), we found that the top 5 targets are TNF, PTGS2, AKT1, IL1β, and GAPDH. They may be potential core targets, suggesting their potential significance in the pharmacological process.

#### 2.2.2. GO Enrichment and KEGG Pathway Analysis

To understand the complex interactions among Chinese patent medicines, ingredients, diseases, and corresponding targets, an ‘active ingredient-targets-pathways’ network map was established by Cyto scape. In this study, the compounds eleutheroside E, quercetin, betaine, sucrose, chlorogenic acid, isoquercitrin, 3,5-Dicaffeoylquinic acid, oleanonic acid, 5-hydroxymethylfurfural, α-linolenic acid, quinic acid, ursolic acid, and protocatechuic acid were found to interact with target regulatory pathways. These interactions may represent key active ingredients of ASFEE in the prevention of AKI (Figure 1D). The interaction network diagram illustrating the relationships among active ingredients, targets, and pathways highlights the potential of ASFEE in preventing AKI.

To further clarify the biological functions of the identified cross-targets, a Gene Ontology (GO) enrichment analysis was performed. The GO analysis identified the top ten biological processes associated with ASFEE in relation to AKI, which include responses to drugs, reactive oxygen species metabolic processes, membrane rafts, membrane microdomains, postsynaptic neurotransmitter receptor activity, and neurotransmitter receptor activity among others (Figure 1E). KEGG analysis revealed that the differentially expressed genes were primarily enriched in MAPK, p53, NF-κB, TNF, IL-17 and PI3K-AKT signaling pathways (Figure 1F). It is suggested that the main mechanism by which ASFEE prevents AKI may be related to the above-mentioned signaling pathways.

#### 2.2.3. Molecular Docking Analysis

Binding energy serves as an indicator for assessing the compatibility between small molecules and proteins, as lower binding energy corresponds to higher binding stability. The results demonstrate that the binding energies between Eleutheroside E and the p65-p50 heterodimer (PDB: 1VKX) and IL-17 (PDB: 4HR9) are −6.6 and −7.8 kcal/mol, respectively. Similarly, the binding energies between quercetin and the p65-p50 heterodimer (PDB: 1VKX) and IL-17 (PDB: 4HR9) are −7.2 and −6.9 kcal/mol, respectively (Figure 1G–J). To further elucidate the potential synergistic effects of the compounds under investigation, their binding energies were compared with those of previously reported inhibitors targeting the p65-p50 heterodimer (PDB: 1VKX). Specifically, a series of novel pyxinol derivatives (compounds 2a–2h) have been reported to exhibit binding energies ranging from −7.42 to −9.38 kcal/mol [13]. In the present study, Eleutheroside E and quercetin demonstrated binding energies of −6.6 kcal/mol and −7.2 kcal/mol, respectively. These values are comparable to those of compounds 2a (−7.71 kcal/mol), 2c (−7.98 kcal/mol), 2d (−7.44 kcal/mol), 2e (−7.49 kcal/mol), 2f (−7.42 kcal/mol), 2g (−7.56 kcal/mol), and 2h (−7.53 kcal/mol). For the IL-17 target (PDB: 4HR9), quercetin and Eleutheroside E exhibited binding energies of −6.9 kcal/mol and −7.8 kcal/mol, respectively, which are significantly lower than those of paeoniflorin (−5.63 kcal/mol) and catechin (−5.58 kcal/mol) [14]. It should be noted that a lower (more negative) binding energy corresponds to greater binding stability. These findings align with the analysis of the KEGG pathway, further confirming the crucial contribution of the NF-κB and IL-17 signaling pathways in the curative effects of ASFEE on AKI.

### 2.3. The Effect of ASFEE on DDP-Induced HK-2 Cell Injury

**Figure 2:**
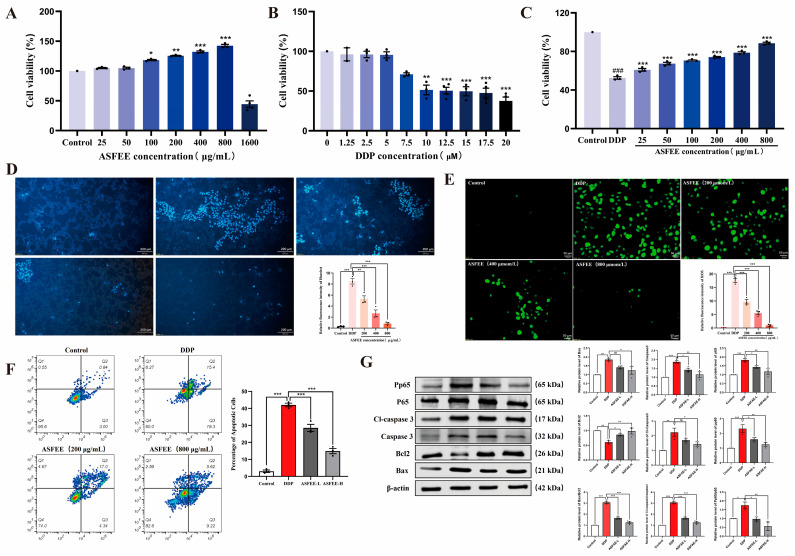
Determination of the protective effect of ASFEE against DDP-induced HK-2 cell injury. (**A**) Effects of different ASFEE concentrations on HK-2 cell viability; (**B**) Establishment of an acute injury model in DDP-induced HK-2 Cells; (**C**) The protective effect of ASFEE pretreatment on HK-2 cell viability in the DDP-induced AKI model; (**D**) Effects of ASFEE on HK-2 cell morphology observed by Hoechst 33258 staining; (**E**) Antioxidant effects of ASFEE against DDP-induced oxidative stress; (**F**) ASFEE inhibits DDP-induced apoptosis in HK-2 cells; (**G**) The effect of ASFEE on Bax, Bcl-2, caspase3, Cleaved-caspase3, p65 and p-p65 proteins in DDP-induced HK-2 cell injury. ### *p* < 0.001 vs. control group; * *p* < 0.05, ** *p* < 0.01, *** *p* < 0.001 vs. DDP group, ns means not significant.

The results of the CCK8 assay indicate that within the concentration range of 25 to 800 μg/mL and the viability of HK-2 cells was maintained at ≥100%. At a concentration of 800 μg/mL, the viability in the ASFEE group exhibited an increase of 43 ± 6.63% compared to the control group. However, at a concentration of 1600 μg/mL, there was a significant decrease in the viability within the ASFEE treatment group, dropping below 60% (Figure 2A). Consequently, this study selected ASFEE concentrations of 200, 400, and 800 μg/mL as low, medium, and high concentration groups for subsequent experiments. As illustrated in Figure 2B, an increase in DDP concentration was positively correlated with a decrease in the cell viability. At a concentration of 17.5 μM, the cell viability decreased to 50–60% (*p* < 0.0001), indicating that the DDP-induced AKI model was effectively established. Consequently, a DDP concentration of 17.5 μM was selected for subsequent experimental procedures. According to Figure 2C, when the ASFEE concentration was set at 200 μg/mL, 400 μg/mL, and 800 μg/mL, the viability of HK-2 cells were 60.03 ± 3.08%, 68.97 ± 3.69%, and 86.19 ± 2.88%, respectively. These values represented significant improvements compared to the DDP group (*p* < 0.001), and a positive correlation between ASFEE concentration and cell viability was observed. The experimental findings demonstrated that pretreatment with varying concentrations of ASFEE (200 μg/mL, 400 μg/mL, and 800 μg/mL) significantly enhanced the viability of injured HK-2 cells.

Hoechst 33258 (Figure 2D) staining revealed a regular morphology in the control HK-2 cells, with nuclei showing round or oval blue fluorescence, without apparent abnormalities. In the DDP group (17.5 μM), the nuclei displayed typical apoptotic features, such as chromatin condensation (manifested as high-intensity fluorescent spots) and nuclear fragmentation (appearing as fluorescent granules), with a significantly increased proportion of apoptotic cells (approximately 50%). In the ASFEE pretreatment groups (200–800 μg/mL), the nuclear morphological damage was progressively alleviated with increasing extract concentration. Notably, in the 800 μg/mL group, the nuclear morphology was nearly comparable to that of the control group, with only a few cells exhibiting mild shrinkage. The percentage of apoptotic cells was significantly reduced compared to the DDP group (*p* < 0.05), suggesting that ASFEE may exert a protective effect against DDP-induced apoptosis in HK-2 cells by preserving nuclear integrity. Figure 2E presents the results of ROS fluorescence staining. The data revealed that the ROS level in the model group was markedly elevated compared to the control group (*p* < 0.0001). When compared to the model group, the ASFEE treatment groups (200 μg/mL, 400 μg/mL, and 800 μg/mL) showed a significant decrease in ROS levels (*p* < 0.001). These findings indicate that ASFEE at various concentrations can effectively mitigate the DDP-induced elevation of intracellular ROS, suggesting that ASFEE may ameliorate acute DDP-induced cell injury through the regulation of oxidative stress.

The flow cytometry results (Figure 2F) demonstrated that, compared with control group, the apoptosis rate of HK-2 cells in the DDP group was 42.0 ± 1.9%. In comparison with DDP group, the apoptosis rate of HK-2 cells in the ASFEE groups treated with different concentrations (200 and 800 μg/mL) decreased as the dosage increased. These values were 28.5 ± 3.6% and 14.9 ± 2.7%, respectively.

The results of Western blotting indicated that, in comparison to control group, the expression levels of Bax, caspase3, Cleaved-caspase3, p65, and p-p65 in the DDP group exhibited a significant upward trend, with relative expression levels increasing (*p* < 0.01). Conversely, the expression level of Bcl-2 demonstrated a downward trend, reflecting a relative decrease in its expression (*p* < 0.01). When compared to DDP group, ASFEE was found to downregulate the expression levels of Bax, caspase3, Cleaved-caspase3, p65, and p-p65 (*p* < 0.05; *p* < 0.01), while simultaneously up regulating the expression level of Bcl-2 (*p* < 0.05; *p* < 0.01) in a dose dependent manner (Figure 2G).

### 2.4. Analysis of Serum Biochemical Indicators and Kidney Histopathology Features

**Figure 3:**
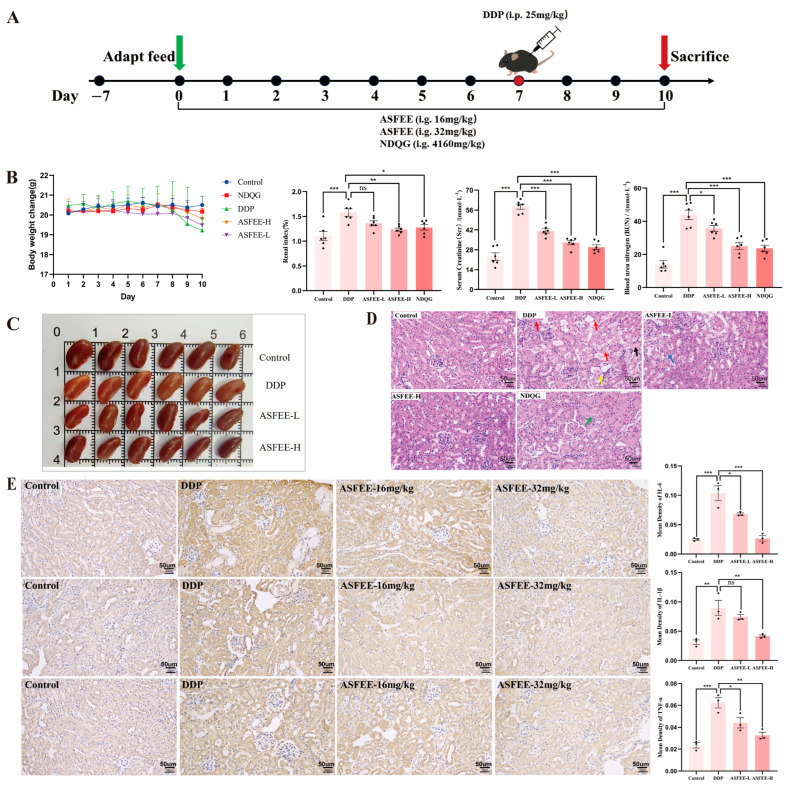
Determination of the protective effect of ASFEE against DDP-induced AKI. (**A**) Schematic diagram of the mouse experiment; (**B**) Body weight changes, kidney index, Scr and BUN levels were measured (*n* = 6); (**C**) Kidney tissue of mice; (**D**) kidney tissue morphology changed by H&E staining, the red arrows indicate partial necrosis and degeneration of the epithelial cells, with unclear cell contours and fragmented nuclei, yellow arrows indicate partial renal tubular abscesses, with a large amount of necrotic cell debris in the lumen, the black arrow indicates that the epithelial cells in the renal tubules have atrophied, and the lumen is filled with protein mucus, the blue arrows indicate partial dilation of renal tubules, edema of epithelial cells, loose cytoplasm and unclear contours, the green arrow indicates that the renal tubular epithelial cells are edematous, with loose cytoplasm and unclear contours; (**E**) Immunohistochemical detection of IL-6, IL-1β and TNF-α levels in mice kidney tissues. * *p* < 0.05, ** *p* < 0.01, *** *p* < 0.001 vs. DDP group, ns means not significant.

Animal experiments were meticulously designed and conducted (Figure 3A) to assess the protective effects of ASFEE on AKI. Figure 3B illustrate the changes in body weight and kidney index for each group of mice. Scr and blood urea nitrogen (BUN) are critical biomarkers of kidney function, serving as indicators of the extent of kidney damage. The research findings demonstrated that the levels of Scr and BUN were significantly elevated following the administration of DDP. However, treatment with both doses of ASFEE resulted in a notable reduction in the levels of Scr and BUN. Figure 3C illustrates the kidney tissues across different experimental groups. Notably, the kidneys in the DDP group exhibited a whitening effect, whereas the ASFEE pretreatment group demonstrated a significant improvement in tissue coloration. Histopathological analysis of Hematoxylin and eosin (H&E) -stained kidney tissue sections provides clear morphological evidence of DDP -induced kidney injury and the protective role of ASFEE. In DDP-treated mice, notable inflammatory cell infiltration and focal necrosis were observed in the kidney parenchyma, whereas ASFEE administration significantly attenuated these pathological changes (Figure 3D). To further elucidate the impact of ASFEE on DDP -induced inflammation in mice, we examined the alterations in kidney inflammatory markers across different experimental groups. As compared to the control group, the levels of pro-inflammatory cytokines, including IL-1β, IL-6, and TNF-α, were markedly elevated in the DDP group. Administration of ASFEE significantly attenuated these increases (*p* < 0.05), as illustrated in Figure 3E.

### 2.5. Transcriptome Analysis in Kidney Tissue

**Figure 4:**
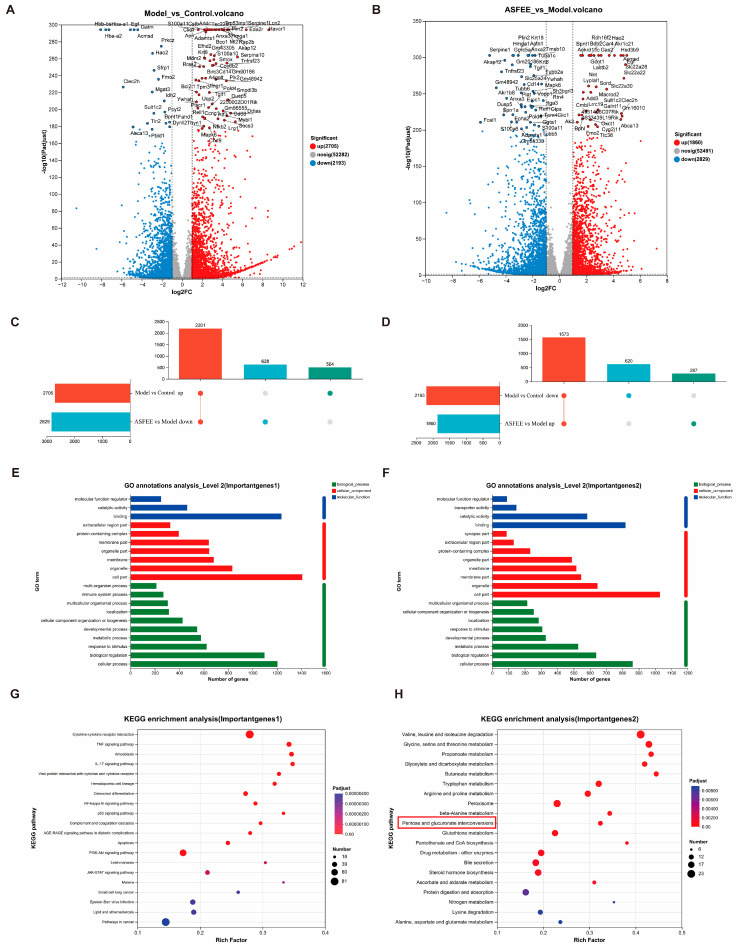
Differentially expressed genes in kidney tissue based on RNA-seq analysis. (**A**) Volcano plot of DEGs of Model vs. Control; (**B**) Volcano plot of DEGs of ASFEE vs. Model; (**C**) upset plot of DEGs downregulated by ASFEE vs. model and upregulated by model vs. control; (**D**) upset plot of DEGs upregulated by ASFEE vs. model and downregulated by model vs. control; (**E**) GO functional annotation characterization of the intersection genes between the downregulated genes in the ASFEE vs. model comparison and the upregulated genes in the model vs. control comparison; (**F**) GO functional annotation characterization of the intersection genes between the upregulated genes in the ASFEE vs. model comparison and the downregulated genes in the model vs. control comparison; (**G**) KEGG enrichment analysis of the intersection genes between the downregulated genes in the ASFEE vs. model comparison and the upregulated genes in the model vs. control comparison; (**H**) KEGG enrichment analysis of the intersection genes between the upregulated genes in the ASFEE vs. model comparison and the downregulated genes in the model vs. control comparison.

A total of 18 samples were subjected to RNA sequencing analysis, with six samples allocated to each of the control group, DDP group, and DDP + ASFEE (32 mg/kg) group. A total of 125.95 Gb of clean data were generated, with each sample yielding more than 5.99 Gb of clean data. Additionally, the proportion of Q30 bases exceeded 96.49%. As illustrated in Figure 4A, a total of 4898 differentially expressed genes (DEGs) were identified between the DDP group and control group, comprising 2705 upregulated and 2193 downregulated genes. As shown in Figure 4B, 4689 DEGs were detected between the DDP + ASFEE (32 mg/kg) group and the DDP group, including 1860 upregulated and 2829 downregulated genes. It can be seen that the administration of ASFEE partially reversed the gene expression changes observed in the DDP group, restoring them toward the levels seen in control group. Importantly, ASFEE mitigates DDP-induced alterations in gene expression associated with AKI. For sequencing data statistics, please refer to Table S3, and for comparisons with reference genomes, please refer to Table S4 in the Supplementary Materials.

To enhance the understanding of the regulatory mechanisms of ASFEE on differentially expressed genes (DEGs) in the kidneys, we conducted functional annotations of DEGs across experimental groups. As illustrated in the Upset plots in Figure 4C,D, administration of ASFEE (32 mg/kg) resulted in the downregulation of 2201 genes in the DDP group compared to control group. Furthermore, ASFEE (32 mg/kg) upregulated 1573 genes that were previously downregulated in the DDP group relative to the control group. These results provide a foundation for identifying a new set of target genes modulated by ASFEE prior to performing GO functional annotation analysis. In terms of molecular function, DEGs were predominantly enriched in molecular function regulators, catalytic activity, and binding functions. In terms of cellular components, DEGs were primarily enriched in the extracellular region part, protein-containing complexes, and membrane parts. In the biological process category, DEGs were mainly associated with extracellular region parts, protein-containing complexes, and membrane parts. A comprehensive list of GO terms is provided in Tables S5 and S6. Additionally, KEGG pathway enrichment analysis was performed on the DEGs (upregulated by DDP and downregulated by ASFEE), with results displayed in Figure 4G. Our analysis yielded a total of 316 signaling pathways, encompassing key pathways such as TNF, IL-17, NF-κB, p53, and PI3K-Akt. Further analysis of these pathways indicated that the DEGs regulated by ASFEE were predominantly associated with inflammatory signaling pathways. For example, the MAPK pathway serves as a critical module linking intracellular and extracellular responses, mediates the transmission of extracellular signals into cells, and regulates cellular processes such as growth, differentiation, and inflammation. This may also explain some of the observed results in the GO enrichment analysis. Moreover, TNF and NF-κB exhibit a closely interrelated signaling axis. TNF-α can induce the activation of NF-κB, thereby promoting the production of various inflammatory cytokines. This suggests that ASFEE may exert protective effects against DDP-induced kidney injury in mice by suppressing inflammatory responses. Similarly, Figure 4H demonstrates that the expression of DEG was downregulated by DDP treatment and subsequently upregulated by ASFEE in the metabolic pathways of “valine, leucine and isoleucine degradation,” “glycine, serine and threonine metabolism,” “propanoate metabolism,” and “glyoxylate and dicarboxylate metabolism.” Additionally, it was significantly enriched in the pathway associated with “ascorbate and aldarate metabolism.” These findings suggest that ASFEE may ameliorate DDP-induced kidney injury through the modulation of drug metabolism-related pathways. Similarly, Figure 4H presents the KEGG pathway analysis of DEGs that were downregulated by DDP and upregulated by ASFEE. The results indicate that these differentially expressed genes are significantly enriched in the “valine, leucine and isoleucine degradation” and “glycine, serine and threonine metabolism” pathways. Additionally, the pathways related to “propanoate metabolism,” “glyoxylate and dicarboxylate metabolism,” and “ascorbate and aldarate metabolism” were significantly enriched. These findings suggest that ASFEE may alleviate DDP-induced kidney injury by modulating drug metabolism-related pathways.

### 2.6. Effect of ASFEE on Kidney Inflammation-Related Pathways

**Figure 5:**
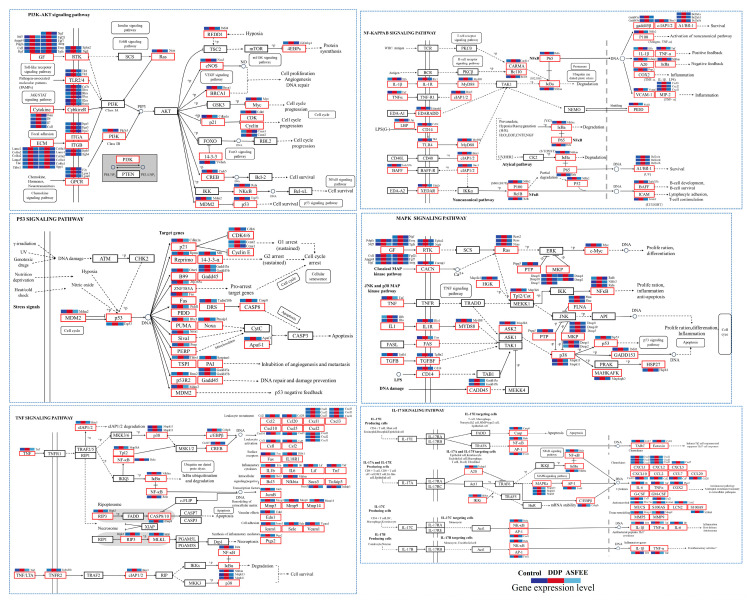
The key regulatory roles of ASFEE on AKI response.

Analysis confirmed significant enrichment of multiple inflammation-related pathways, particularly PI3K-AKT (mmu04151), NF-κB (mmu04064), P53 (mmu04115), MAPK (mmu04010), TNF (mmu04668), and IL-17 (mmu04657) signaling pathways. To further explore the mechanism by which ASFEE influences AKI, we constructed enriched pathway diagrams and heatmaps. Relative to controls, the upregulation of key genes in inflammation-related metabolic pathways indicated that excessive DDP induction triggers kidney inflammation. In contrast, ASFEE group markedly suppressed the expression of these genes, indicating that ASFEE possesses potential protective effects in inhibiting kidney inflammatory responses (Figure 5).

Notably, the regulated genes within these pathways exhibit distinct functional characteristics. Specifically, the 47 differentially expressed genes in the TNF signaling pathway predominantly include pro-inflammatory cytokines (e.g., TNF, IL-1β), chemokines (e.g., Cxcl family, Ccl family), molecules associated with apoptosis and necrosis (e.g., Casp family, Mlkl), as well as transcriptional regulators (e.g., Rela, Fos). The 34 genes identified in the p53 signaling pathway are primarily focused on cell cycle regulatory factors (e.g., Ccnb family, Cdk family), apoptotic molecules (e.g., Bax, Bbc3), and genes involved in DNA damage repair mechanisms (e.g., Gadd45 family). In the NF-κB signaling pathway, which comprises 37 genes, there is a concentration on NF-κB family members (e.g., Rela, Relb), inflammatory receptors and adaptors (e.g., Tlr4, Myd88), along with adhesion molecules (e.g., Icam1, Vcam1). The IL-17 signaling pathway encompasses 34 genes that mainly pertain to inflammatory cytokines (e.g., IL-6, TNF), chemokines from the Cxcl family, and MAPK kinases such as Mapk11 and Mapk13. The PI3K-Akt signaling pathway includes 82 genes that cover growth factor receptors (e.g., Pdgfra; Fgf family), cytokine receptors from the Il family, and molecules that regulate cell survival processes such as Bcl2l1. Lastly, within the MAPK signaling pathway consisting of 67 genes predominately characterized by upstream MAPK kinases (e.g., Map3k family), inflammatory signaling molecules like Il1r1 are present alongside transcriptional regulators including Myc and Fos.

Importantly, these pathways do not operate in isolation; rather, they form an interconnected network characterized by complex cross-regulation, collectively contributing to DDP-induced kidney inflammation and the progression of AKI.

### 2.7. Metabolomic Analysis in Kidney Tissue

**Figure 6:**
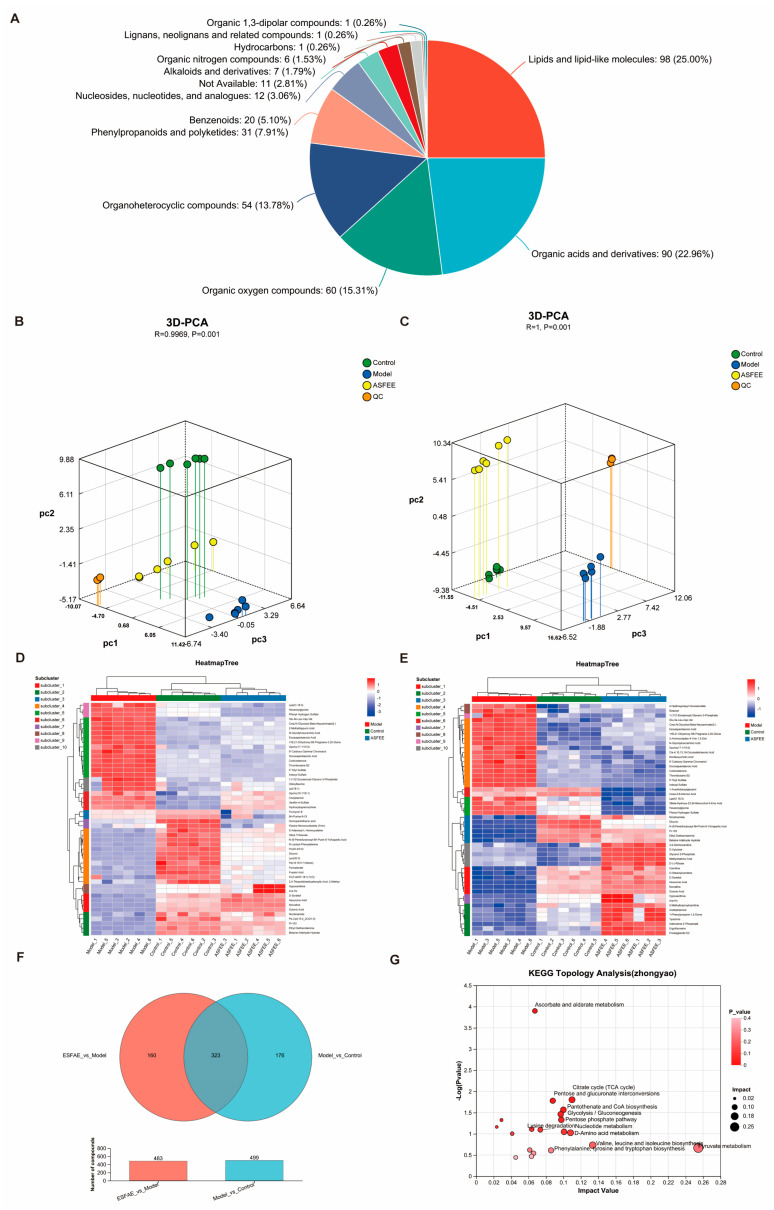
Analysis of differential metabolites in kidney tissue based on metabolomics analysis. (**A**) HMDB-based classification of metabolite categories; (**B**) 3D-PCA analysis of positive ion mode metabolites; (**C**) 3D-PCA analysis of negative ion mode metabolites; (**D**) clustered heatmap visualization of DAMs of model vs. control; (**E**) heatmap clustering demonstrating DAMs of ASFEE vs. model; (**F**) Venn diagram of DAMs between ASFEE vs. model and model vs. control; (**G**) KEGG topology analysis of intersection metabolite between ASFEE vs. model and model vs. control.

Metabolomic analysis was conducted on the kidney tissues of the control, DDP, and DDP + ASFEE (32 mg/kg) groups (n = 6). The total ion chromatograms in the positive and negative ion modes for each group are shown in Figures S3–S8. Figure 6A illustrates the classification of metabolites, revealing a total of 13 identified substances, including lipids and lipid-like molecules, organic acids and derivatives, organic oxygen compounds, organoheterocyclic compounds, benzenoids, phenylpropanoids and polyketides, among others. Table S7 provides the total ion count and identification statistics. Principal component analysis (3D-PCA) was conducted in both positive and negative ion modes on samples from control group, DDP group, and DDP + ASFEE (32 mg/kg) group. The results are presented in Figure 6B,C. The analysis reveals a distinct separation among the three groups, especially between the model (DDP) and control groups. The complete differentiation of the DDP + ASFEE (32 mg/kg) group further confirms the successful establishment of the model. To further elucidate the underlying mechanism of ASFEE’s protective effect against DDP-induced kidney injury, we conducted a comparative analysis by identifying the intersection of differentially expressed metabolites between the ASFEE vs. Control group and the DDP vs. Control group, resulting in a total of 323 differential metabolites. Subsequently, cluster analysis was performed to visualize the accumulation patterns of these differentially accumulated metabolites (DAMs) across the various experimental groups, with the results presented in Figure 6D,E. Figure 6F presents the Venn diagram of DAMs derived from the comparisons of ASFEE vs. DDP and DDP vs. control. A total of 323 overlapping metabolites were identified, which may represent key metabolites through which ASFEE exerts its protective effects against AKI (Table S7). Furthermore, pathway enrichment analysis was carried out as illustrated in Figure 6G and Table S8, these metabolites are involved in several metabolic pathways, including ascorbate and aldarate metabolism, linoleic acid metabolism, the citrate cycle (TCA cycle), pentose and glucuronate interconversions, pantothenate and CoA biosynthesis, glycolysis/gluconeogenesis, D-amino acid metabolism, as well as the biosynthesis of valine, leucine, and isoleucine.

### 2.8. Integrated Analysis of ASFEE Protection Against DDP-Induced AKI

**Figure 7:**
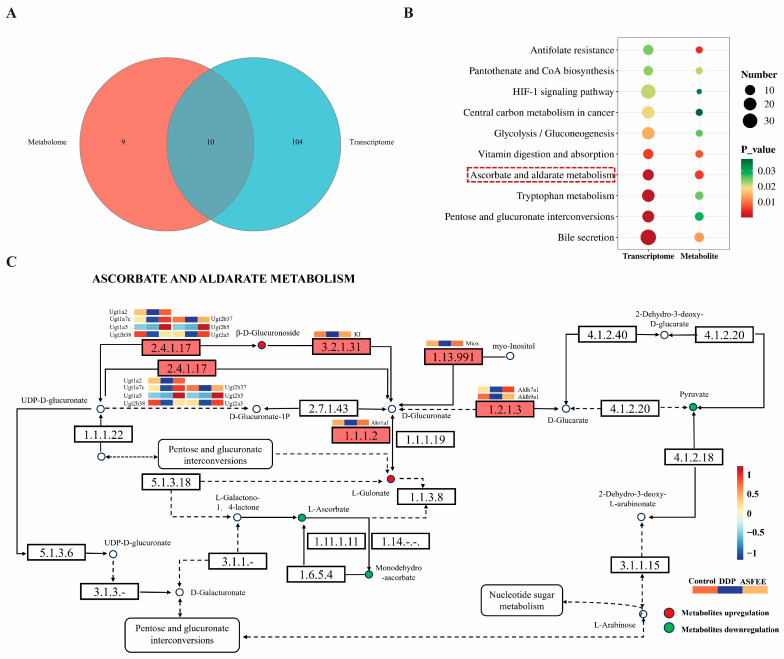
Combined analysis of metabolomics and transcriptomics. (**A**) Venn diagrams of the metabolome and transcriptome; (**B**) Bubble map of important metabolic pathways in the metabolome-transcriptome; (**C**) ASFEE protects against DDP-induced AKI by ascorbate and aldarate metabolism.

To uncover the mechanism by which ASFEE protects against AKI, we investigated its effects on gene and metabolite expression profiles. An integrated metabolomics and transcriptomics approach was employed to gain deeper insights into the associated molecular pathways. A total of 10 common KEGG pathways were identified through integrated metabolomics and transcriptomics analysis (Figure 7A). These pathways included glycolysis/gluconeogenesis (mmu00010), antifolate resistance (mmu01523), vitamin digestion and absorption (mmu04977), HIF-1 signaling pathway (mmu04066), ascorbate and aldarate metabolism (mmu00053), pantothenate and CoA biosynthesis (mmu00770), and tryptophan metabolism (mmu00380) (Figure 7B). Among these, the ascorbate and aldarate metabolism pathway exhibited the lowest *p*-value and was the most significantly enriched in both the metabolomic and transcriptomic datasets. Further analysis of the differential metabolites and genes within this pathway revealed the specific regulatory effects of ASFEE on DDP-induced AKI, as illustrated in Figure 7C. Specifically, ASFEE exerts anti-AKI effects by regulating six metabolites and 13 genes in the ascorbic acid and aldose metabolic pathways. In terms of metabolites, it reverses the downregulation of ascorbic acid caused by DDP to enhance antioxidant capacity, reduces the level of glucuronic acid conjugates to promote detoxification, and restores the levels of guluronic acid and pyruvate to support energy and ascorbic acid synthesis. At the transcriptional level, 11 out of the 13 genes belong to the UGT family and related enzyme genes. ASFEE can restore the UGT expression disorder caused by DDP, enhance the detoxification function of the kidneys, and simultaneously regulate the genes of aldehyde dehydrogenase and aldehyde-ketone reductase to maintain metabolic homeostasis. In summary, ASFEE functions by enhancing antioxidation, promoting UGT-mediated detoxification, and restoring key metabolic intermediates.

### 2.9. Validation of Key Genes and Proteins

**Figure 8:**
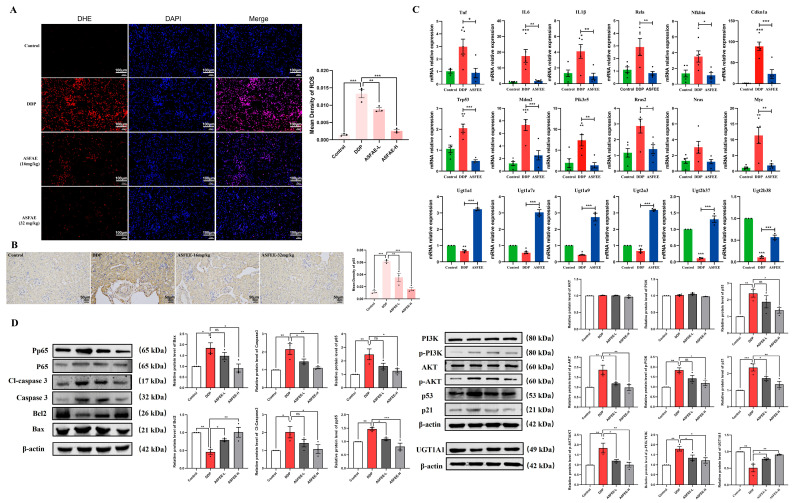
Verification results of relevant genes and proteins. (**A**) Fluorescence detection of ROS in kidney tissues of DDP-induced AKI mice by ASFEE; (**B**) The effect of ASFEE on p65 protein in kidney tissue of mice with DDP-induced by immunohistochemical detection; (**C**) A qRT-PCR-based analysis was conducted to assess the expression levels of 18 selected genes; (**D**) The protein expressions and Quantitative results of p-p65, p65, caspase3, Cleaved-caspase3, Bcl2, Bax, PI3K, p-PI3K, p53, p21 and UGT1A1 by Western blot. * *p* < 0.05, ** *p* < 0.01, *** *p* < 0.001 vs. DDP group. ns means not significant.

To further clarify the key factors in the protective mechanism of ASFEE against DDP-induced AKI, we detected the ROS levels in the kidney tissues of mice. Compared with control group, the ROS levels in the kidney tissues of mice in the DDP group were significantly increased. Compared with t DDP group, ROS levels in the kidneys of mice in different ASFEE intervention groups decreased in a dose-dependent manner, as shown in Figure 8A. The immunohistochemical results showed that the p65 protein presented the same trend among different groups (Figure 8B). To further elucidate the role of ASFEE in AKI by regulating inflammation-related pathways, we evaluated the mRNA levels of TNF, IL-6, IL-1β, Rela (p65), Nfkbia, Cdkn1a (p21), Trp53, Mdm2, Pik3r 5, Rras2, Nras and Myc in the kidney tissues of mice in each experimental group by RT-qPCR method. The findings showed that the expression of these genes in DDP group was significantly increased compared with control group. On the contrary, ASFEE treatment led to a significantly reduced the expression of these genes. In addition, the effect of ASFEE on the ascorbate and aldarate metabolism pathways was evaluated by the same method. The mRNA levels of Ugt1a1, Ugt1a7c, Ugt1a9, Ugt2a3, Ugt2b37 and Ugt2b38 were detected. Compared with control group, the expression of these genes in the DDP group was significantly decreased. In contrast, ASFEE treatment led to a significant increase in the expression of these genes (Figure 8C). Furthermore, Western blot analysis demonstrated that DDP significantly elevated the expression levels of inflammatory markers, including p65, p-p65, caspase-3, cleaved caspase-3, Bax/Bcl-2 ratio, p53, p21, PI3K, and p-PI3K. In contrast, ASFEE treatment markedly reduced the expression of these proteins (Figure 8D). Notably, DDP significantly downregulated UGT1A1 expression, whereas ASFEE treatment restored its protein levels, indicating an involvement of ASFEE in the ascorbate and aldarate metabolism pathway. To further verify the function and necessity of UGT1A1, the UGT1A1 inhibitor 1,2,3, 6-tetraenylglucose (TeGG) was used in HK-2 cells. The RT-qPCR results showed that TeGG could upregulate the mRNA expression of BAX and downregulate the expression of Bcl-2. Meanwhile, the mRNA levels of IL-6, IL-1β and TNF were upregulated. However, the TeGG+ASFEE intervention group was able to reverse the above phenomenon (Figure S9). In addition, to explore the crosstalk between PI3K/Akt and NF-κB signaling, we used the PI3K/Akt inhibitor Sophocarpine in the HK-2 cell model and detected the gene levels of NFKBIA and RELA by RT-qPCR. The results indicated that both Sophocarpine administration and ASFEE administration could downregulate the increased expression of NFKBIA and RELA (P65) induced by DDP (Figure S10). These findings suggest that ASFEE may exert a protective effect against DPP-induced AKI by upregulating UGT1A1 and inhibiting PI3K/Akt to reduce inflammation.

## 3. Discussion

As a widely used chemotherapy drug in clinical practice, the dose-limiting nephrotoxicity of DDP is the key issue restricting its efficacy. Exploring safe and effective protective strategies has significant clinical significance [15]. This study is the first to systematically confirm that ASFEE has a significant protective effect on DDP-induced AKI. Through in vitro and in vivo experiments combined with multi-omics analysis, it reveals that the core mechanism is to regulate UGT-mediated Ascorbate and aldarate metabolism. Furthermore, it collaboratively regulates multiple signaling pathways such as NF-κB, PI3K-Akt and IL-17.

In vitro experiments confirmed that ASFEE can enhance the viability of DDP-induced HK-2 cells and inhibit apoptosis, suggesting the protective effect of ASFEE on kidney cells. In vivo experiment, ASFEE pretreatment can significantly improve kidney function in mice caused by DDP index (Scr, BUN) and the kidney pathological damage (kidney tubular necrosis, inflammatory infiltration), omics analysis further pointing at the metabolic level key changes: After DDP treatment, the metabolism of Ascorbate and aldarate in the kidneys of mice was significantly disrupted, while ASFEE could reverse this trend, especially by upregulating the expression of UGT family members. UGT enzymes are crucial for detoxifying exogenous substances (e.g., narcotics) and metabolizing endogenous compounds such as bilirubin, bile and fatty acids. This dual protective function not only safeguards the organism from harmful chemicals but also modulates the activity of key endogenous mediators involved in cellular growth and differentiation [16,17]. These mechanisms may represent the initial pathway through which ASFEE alleviates DDP toxicity. Ascorbic acid (vitamin C), as a key metabolite in this pathway, exhibits potent antioxidant, anti-inflammatory, and immunomodulatory properties [18]. Studies have shown that vitamin C plays a significant role in preventing and mitigating the severity of various viral and bacterial infections [19]. It can alleviate damage to various organs by reducing oxidative stress [20]. The restoration of its level may enhance the antioxidant capacity of the kidneys and reduce the accumulation of ROS induced by DDP. The balance of uronate metabolism helps to reduce kidney tubular injury [21].

It is worth noting that the regulation of metabolic pathways does not act in isolation but forms a synergistic effect with inflammatory and apoptosis-related signaling pathways. Inflammation caused by NF-κB p65 is also highly involved in the development of AKI. p-p65 is a transcription factor located downstream of the NF-κB signaling pathway. In the case of AKI, a large amount of mediators will be released, leading to kidney tissue destruction [22]. After AKI, the inflammatory factors and kidney damage molecules in mice increase significantly. AKI can induce inflammatory responses and cause damage to the body. Our results showed that the levels of NF- κB p65, TNF-α, IL-6 and IL-1β in the kidneys of mice increased after DDP injection. Consistent with this finding, an increasing amount of evidence has demonstrated that NF-κB p65 is activated in the DDP -induced nephrotoxicity model and stimulates the production of various pro-inflammatory cytokines [23,24]. However, ASFEE treatment alleviated these inflammatory cytokines, indicating that ASFEE can inhibit NF-κB signaling pathway-mediated inflammatory damage and protect kidney tissue in a dose-dependent manner.

The PI3K/Akt signaling pathway plays a crucial role in cell viability, metabolism, migration and proliferation [15]. Accumulating evidence indicates the PI3K/Akt signaling pathway is a key therapeutic target in AKI [25,26]. PI3K is a phosphatidylinositol kinase, which possesses the activity of serine/threonine-specific protein kinase and phosphatidylinositol kinase [27]. After activation, members of the phosphatidylinositol family on the cell membrane can be phosphorylated, and the downstream signaling molecule Akt can be activated. The PI3K/AKT pathway acts as an upstream regulator of the NF-κB pathway. Many compounds can activate the NF-κB pathway through PI3K/AKT, protecting cells or tissues from damage [28]. In this study, ASFEE treatment significantly suppressed the DDP-induced phosphory-lation of Akt, as evidenced by reduced p-Akt levels. The attenuation of these signaling cascades resulted in reduced expression of pro-apoptotic factors (Bax and Caspase-3) while promoting cell survival genes (Bcl-2), ultimately ameliorating kidney tubular epithelial cell apoptosis and excessive inflammatory responses. Therefore, ASFEE exerts its protective effects against DDP-induced AKI primarily through suppression of the PI3K/Akt-NF-κB signaling axis.

IL-17 mainly plays a role in coordinating various inflammatory and immune responses and is related to the pathogenesis of many diseases [29]. It can trigger multiple downstream pathways, such as the NF-κB pathway, which plays a key role in regulating inflammatory responses [30]. In addition, IL-17 has been identified as an important factor in the development of kidney diseases [31]. Our results indicate that ASFEE can effectively inhibit the activation of the IL-17 signaling pathway and block the conduction of key inflammatory pathways such as downstream NF-κB. The synergistic regulation of these pathways may be a manifestation of the cascading effect of ASFEE from “metabolic repair” to “inflammatory suppression” and then to “cellular protection”. The improvement of metabolic pathways mediated by UGT provides a metabolic basis for upstream regulation and the balance of downstream signaling pathways, while the synergy of multiple signaling pathways amplifies the protective effect.

This study demonstrated that ASFEE exerts a significant protective effect on cisplatin-induced nephrotoxicity through a multi-target mechanism. Its notable feature is that while regulating the PI3K/Akt pathway and inhibiting inflammatory and apoptotic responses, it can significantly upregulate UGT1A1. This combined mechanism of action distinguishes ASFEE from other natural products that have been reported to have renal protective activity. For instance, both protopanaxadiol and baicalein have been proven to improve cisplatin-induced acute kidney injury, but their effects are mainly limited to different ferroptosis pathways—targeting GPX4 and ALOX12, respectively [32,33]. In contrast, the scope of application of ASFEE is much broader. In addition to its effective regulation of cell survival and death pathways, a key advantage of ASFEE lies in its unique impact on UGT1A1. Unlike known UGT1A1 inhibitors such as silybin or microRNA regulators (such as miR-141-3p) [34,35], which typically inhibit the activity or expression of this enzyme, ASFEE, as a positive regulator, can significantly increase the level of UGT1A1. This induction effect is likely to promote the metabolic clearance of cisplatin and its toxic metabolites, providing a detoxification strategy that is not available in comparison with ferroptosis inhibitors. Therefore, the innovation of ASFEE lies in its ability to integrate “detoxification by upregulating UGT1A1”, “cell protection by inhibiting PI3K/Akt”, and “anti-inflammatory/anti-apoptotic” effects, proposing a new multi-level and synergistic treatment strategy for cisplatin-induced acute kidney injury.

## 4. Materials and Methods

### 4.1. Sample Preparation

*Acanthopanax senticosus* (*Rupr.et Maxim.*) *Fruit* (ASF) (202208010) was purchased from Jilin Guo’an Pharmaceutical Co., Ltd. (Jilin, China). A total of 200 g of ASF was subjected to ultrasonic extraction three times with 75% ethanol at a solvent-to-sample ratio of 10:1 (*v*/*w*) for 1 h each time under conditions of 100 Hz and 30 °C. 75% ethanol was chosen because it has been widely proven to effectively extract polar active components (such as flavonoids and saponins) from *Acanthopanax senticosus* plants [36]. The adopted material-liquid ratio is 1:10 (*w*/*v*), which is an optimization result based on the pre-experiment to ensure the maximization of extraction efficiency. The resulting extracts were filtered, and the filtrates were combined. The combined extracts were concentrated using a rotary evaporator and subsequently freeze-dried to obtain ASFEE, with an extraction yield of 6.83%.

### 4.2. UPLC-MS/MS Analysis

The ASFEE sample was dissolved in 80% methanol. Chromatographic separation was carried out on an ACQUITY UPLC I-Class system (Waters, Milford, MA, USA) equipped with an ACQUITY UPLC HSS T3 column (2.1 × 100 mm, 1.8 µm, Waters, Milford, MA, USA). The column temperature was maintained at 35 °C, the flow rate was set to 0.3 mL min^−1^, and the injection volume was 10 µL. The mobile phase consisted of phase A (deionized water containing 0.1% formic acid) and phase B (acetonitrile containing 0.1% formic acid), and gradient elution was applied over a 70 min runtime (Table S1). Mass spectrometric data were acquired using a Q Exactive Orbitrap high-resolution mass spectrometer (Thermo Fisher Scientific, Waltham, MA, USA) in Full MS-ddMS^2^ mode. Both positive and negative ionization modes were employed, with a mass scanning range of *m*/*z* 100–1200. The MS^1^ resolution was set to 70,000, while the MS^2^ resolution was set to 17,500. The ion source voltage was 3.2 kV. The capillary ion transport tube temperature was maintained at 320 °C, and the auxiliary gas heater temperature was set to 350 °C. The sheath gas flow rate was adjusted to 40 L/min, and the auxiliary gas flow rate was set to 15 L/min. The AGC target was set to 1 × 10^6^, and the Top N parameter was set to 5. The collision energy for triggering the MS^2^ scan was based on stepped normalized collision energy (NCE) values of 30, 40, and 50.

All solvents were purchased from Merck (Darmstadt, Hesse, Germany), and ultrapure water was prepared with a Millipore system.

### 4.3. Network Pharmacology Construction

The metabolite-protein pathway network was constructed using Cytoscape software (version 3.8.2; Cytoscape Consortium, San Diego, CA, USA) to identify core metabolites and their associated proteins. Candidate targets related to acute kidney injury (AKI) were retrieved from multiple databases, including GeneCards (https://www.genecards.org/) (accessed on 17 May 2024), OMIM (https://omim.org/) (accessed on 17 May 2024), the TCMSP database (https://www.tcmsp-e.com/#/database) (accessed on 17 May 2024), and therapeutic target databases such as TTD (http://db.idrblab.net/ttd/) (accessed on 17 May 2024). Preliminary screening of all components was conducted according to oral bioavailability (OB; ≥30%) and drug-likeness (DL) properties (≥0.18) [37]. Potential targets of *Acanthopanax senticosus* fruit were predicted by screening keywords in SwissTargetPrediction (http://www.swisstargetprediction.ch/) (accessed on 17 May 2024), BATMAN-TCM (http://bionet.ncpsb.org/batman-tcm/) (accessed on 17 May 2024), STITCH 5.0 (http://stitch.embl.de/) (accessed on 17 May 2024), and ChEMBL (https://www.ebi.ac.uk/chembl/) (accessed on 17 May 2024). The predicted therapeutic targets of *Acanthopanax senticosus* fruit extract (ASFEE) in AKI treatment were defined as the overlapping genes between drug-related targets and disease-associated targets. Subsequently, KEGG (Kyoto Encyclopedia of Genes and Genomes) pathway and GO enrichment analyses were performed on the potential targets using the Clue GO plugin within Cytoscape.

### 4.4. Molecular Docking

Molecular docking was performed using AutoDock Vina (v1.1.2). Retrieve the protein structures from the protein database (PDB ID: 1VKX and 4HR9) and prepare them using AutoDock Tools (v1.5.6): Remove water molecules and non-protein atoms, add polar hydrogen atoms, assign Gasteiger charges, and use PROPKA (v3.1) to set the protonation state at pH 7.4. The molecular structures of Eleutheroside E (PubChem CID: 71312557) and quercetin (PubChem CID: 5280459) were retrieved from PubChem (https://pubchem.ncbi.nlm.nih.gov/, accessed on 27 May 2025). The ligands obtained from PubChem were converted into three-dimensional structures by Open Babel (v3.1.1), hydrogen atoms, charges and rotatable bonds were defined in AutoDock Tools, and saved in PDBQT format. The docking grid (40 × 40 × 40 Å, spacing 0.375 Å) is centered on the active site (identified by eutectic ligands or CASTp). The Vina parameters are set as: exhaustiveness = 8, num_modes = 10, energy_range = 3 kcal/mol. The binding affinity (ΔG, in kcal/mol) was calculated, and the Ki value was derived through the formula Ki = exp (ΔG/RT) (R = 1.987 cal/mol·K, T = 298 K). The root mean square deviation (RMSD) is ≤2.0 Å through re-docking verification protocol. Visualize the interaction in PyMOL (v2.5).

### 4.5. Cell Culture and Treatment

HK-2 cells were cultured in IMDM and maintained in a 5% CO_2_ incubator at 37 °C, with the culture medium replaced regularly. Cells in the logarithmic growth phase and with a fusion degree reaching 90% were collected and digested with 0.25% trypsin. Cells (approximately 4.5 × 10^3^/well) were seeded into 96-well plates for 24 h to ensure cell adhesion. The ASFEE extract was dissolved in dimethyl sulfoxide (DMSO) to prepare a stock solution, with the final concentration of DMSO in all treatments maintained below 0.1% (*v*/*v*). Cells were pretreated with ASFEE (200, 400, and 800 μg/mL) for 12 h, followed by treatment with 17.5 μM DDP (Solarbio, Lot: 124J021) for additional 12 h. The experiment was independently repeated three times, with six parallel complex Wells set for each experiment (n = 3). Cell viability was detected by the CCK8 method.

### 4.6. Hoechst 33258 Dyeing

The nuclear morphological changes in HK-2 were detected by using the Hoechst 33258 staining kit. Cells were fixed with 4% paraformaldehyde, then, washed with PBS, and permeated with 0.2% Triton X-100. After PBS washing, each well was stained with Hoechst 33258 dye (10 μg/mL) for 5 min. Bright blue fluorescence was observed in the cell nuclei by fluorescence microscopy, and the results were quantified by Image J software (version 1.54p, National Institutes of Health, Bethesda, MD, USA).

### 4.7. Assessment of ROS Levels

The ROS level in HK-2 cells was detected by DCFH-DA (2′, 7′-dichlorofluorooctane diacetate) probe. Cells were incubated with DCFH-DA at a final concentration of 10 μM in the culture medium for 30 min at 37 °C in the dark. After being rinsed twice with PBS, fluorescence intensity was measured and normalized to the total cell number.

### 4.8. Annexin V-FITC/PI Apoptosis Detection

According to the instructions of Annexin V-FITC/PI double staining apoptosis kit, HK-2 cells were digested with trypsin, and the cell precipitate was obtained by centrifugation. The medium was discarded, washed with PBS, and the cell precipitate was resuspended in buffer, FITC Annexin V staining solution was added, and incubated in the dark for 5 min. Subsequently, PI staining solution was added and incubated for 5 min. After staining, PBS was added according to the cell count, and the proportion of apoptotic cells was analyzed by flow cytometry.

### 4.9. Experimental Animals

8-week-old male C57BL/6 mice (n = 30; body weight: 20–25 g) were purchased from Liaoning Changsheng Biotechnology Co., Ltd. (Benxi, China). and housed under specific pathogen free conditions with controlled temperature (22 ± 2 °C), humidity (50 ± 10%), and a 12 h light/dark cycle. After 7 days of acclimation, the mice were randomly divided into 5 groups using a random number table method: Control group, which was raised normally; Model group: Normal feeding for the first 7 days, and a one-time intraperitoneal injection of DDP on the 7th day. Low-dose ASFEE group (16 mg/kg); ASFEE high-dose group (32 mg/kg); DDP+ Niaoduqing Granules group (4160 mg/kg, Consun Pharmaceutical (Horgos) Co., Ltd. (Xinjiang, China), Lot No. 20230442); Except for control group and DDP group, the remaining groups were continuously administered for 11 days, with a 24 h interval between each administration and daily weight measurement. On the 8th day, except for the blank group, mice in the model group and each experimental group were intraperitoneally injected with DDP (25 mg/kg) to establish AKI models [38,39]. Three days later, all the mice were sacrificed and blood samples were collected for subsequent testing. For all animal experiments, investigators were blinded to group allocation during the experiment and when assessing the outcome. All animal experiment procedures were carried out in accordance with the protocol approved by the Ethics Committee of Jilin Medical University (2020-KJT056).

### 4.10. SCr and BUN

Blood was collected 72 h after DDP administration and centrifuged at 4 °C (3000 rpm for 10 min). SCr and BUN were detected, respectively, using creatinine assay kits and urea assay kits (Nanjing Jiancheng Bioengineering Company, Nanjing, China).

### 4.11. H&E Staining and ROS Detection

The kidney tissue was fixed with 4% paraformaldehyde for 24 h, trimmed, dehydrated and transparent with gradient alcohol and xylene, and embedded in paraffin. The section thickness was 4 μm. After spreading and baking, HE staining and ROS staining were carried out.

### 4.12. Immunohistochemistry

The expressions of IL-1β, TNF-α, IL-6 and p65 in kidney tissues were detected by immunohistochemical method. The kidneys were fixed with 4% paraformaldehyde, embedded in paraffin, sectioned, and incubated overnight with IL-1β (1:200), p65 (1:200), IL-6 (1:200), and TNF-α (1:200) primary antibodies at 4 °C. The slices were incubated with horseradish peroxidase (HRP) conjugated secondary antibody.

### 4.13. Total RNA Isolation and Transcriptome Analysis

Total RNA was obtained from kidney tissue using TRIzol^®^ reagent (Invitrogen, Carlsbad, CA, USA). Subsequently, the RNA concentration and integrity were evaluated using Illumina TruSeq™ RNA sample preparation kit (Illumina, San Diego, CA, USA). RNA-seq libraries were constructed and sequenced on the Illumina NovaSeq 6000 platform provided by Shanghai Majorbio Bio-pharm Technology Co., Ltd. (Shanghai, China). The quality of the original data was controlled using FASTP software (Version 0.23.4), and then these clean reads were compared with the reference genome using HISAT2 (Version 2.2.1). The reference genome from Ensembl genome database (http://asia.ensembl.org/Mus_musculus/Info/Index, accessed on 5 March 2025). Gene expression quantification was performed using the transcript per million (TPM) standardized method. Differentially expressed genes (DEGs) were identified based on the criteria of p-adjusted (padj) < 0.05 and |log2 fold-change| > 1 and FDR-adjusted *p*-value < 0.05. GO on in Majorbio cloud platform (https://cloud.majorbio.com/) (http://www.geneontology.org/) URL (accessed on 5 March 2025) and KEGG (http://www.genome.jp/kegg/) URL (accessed on 5 March 2025), data analysis.

### 4.14. Untargeted LC-MS Metabolomics Analysis

90 mg kidney tissue were combined with 250 µL of water. Following this, an 900 µL solution of methanol and acetonitrile, mixed in a 1:1 volume ratio, was introduced, and the resulting mixture underwent ultrasonic treatment at a low temperature. The protein precipitate was obtained through centrifugation at 4 °C for 20 min, after which the supernatant was freeze-dried. An analysis of the metabolites present in the samples was performed using an UHPLC-Q-TOF/MS system. Both positive and negative ions were identified utilizing Electrospray Ionization (ESI) technology. The metabolite structures were ascertained through precise mass matching (<25 ppm) and analysis of secondary spectra. After pretreatment with Pareto-scaling, orthogonal partial least squares discriminant analysis (OPLS-DA) was performed in R (version 3.3.1) (Figure S11) for discriminant analysis, and differentially abundant metabolites were selected based on a variable importance in projection (VIP) value > 1 and a *p*-value < 0.05. For enrichment analyses, metabolic pathways were established using KEGG (http://www.genome.jp/kegg/pathway.html) and tools from the Majorbio Cloud Platform (http://www.omicshare.com/tools), accessed on 5 March 2025.

### 4.15. RT-qPCR

Total RNA was extracted using TRIzol reagent and reverse-transcribed into cDNA. RT-qPCR was carried out with SYBR Green-based detection. Relative mRNA expression was determined via the 2^−ΔΔCt^ method, with normalization to GAPDH. All reactions were run in triplicate. Table S9 contains the primer sequences used in this study.

### 4.16. Western Blot

Total protein was extracted from HK-2 cells or kidney tissues using RIPA lysis buffer (Beyotime, Shanghai, China, Cat# P0013B) containing protease and phosphatase inhibitors. Protein concentration was determined using a BCA protein assay kit (Beyotime, Cat# P0010). Equal amounts of protein (20–30 μg) were separated by 10% SDS-PAGE and transferred to PVDF membranes (Millipore, Darmstadt, Germany, Cat# ISEQ00010). After blocking with 5% non-fat milk in TBST for 1 h at room temperature, the membranes were incubated with specific primary antibodies overnight at 4 °C. The following primary antibodies were used: p-p65 (Proteintech, Wuhan, China, Cat# 82335-1-RR, 1:5000), p65 (Proteintech, Cat# 10745-1-AP, 1:3000), Cleaved Caspase-3 (Proteintech, Cat# 25128-1-AP, 1:1000), Caspase-3 (Proteintech, Cat# 19677-1-AP, 1:1000), Bax (Proteintech, Cat# 60267-1-lg, 1:10,000), Bcl-2 (Proteintech, Cat# 26593-1-AP, 1:1000), p53 (Proteintech, Cat# 10442-1-AP, 1:15,000), p21 (Proteintech, Cat# 10355-1-AP, 1:2000), p-PI3K (Affinity, Cat# AF3241, 1:2000), PI3K (Proteintech, Cat# 60225-1-lg, 1:15,000), p-AKT (Affinity, Cat# AF0016, 1:2000), AKT (Proteintech, Cat# 10176-2-AP, 1:6000), UGT1A1 (Bioswamp, Wuhan, China, Cat# PAB31654, 1:1000), and β-actin (Proteintech, Cat# 66009-1-Ig, 1:25,000). After washing with TBST, the membranes were incubated with HRP-conjugated goat anti-rabbit (Proteintech, Cat# RGAR001, 1:5000) or goat anti-mouse (Proteintech, Cat# RGAM001, 1:5000) secondary antibodies for 1 h at room temperature. Protein bands were visualized using an ECL detection kit (Beyotime, Cat# P0018AFT) and quantified using Image J software (version 1.54p, National Institutes of Health, Bethesda, MD, USA), with β-actin serving as the loading control.

### 4.17. Statistical Analysis

Data are expressed as the mean ± standard deviation from six independent experiments. Statistical comparisons were performed using GraphPad Prism 9.5.0 (GraphPad Software Inc., San Diego, CA, USA) to assess significant differences relative to the corresponding controls in each experimental group. A *p* < 0.05 was defined as statistically significant.

## 5. Conclusions

This study for the first time, through In Vitro and In Vivo experiments combined with multi-omics analysis, systematically confirmed that ASFEE has a significant protective effect on DDP-induced AKI. Research has found that ASFEE regulates the metabolism of ascorbic acid and glucuronic acid mediated by UGT, and collaboratively regulates the NF-κB, PI3K-Akt and IL-17 signaling pathways, forming a multi-target protection mechanism. Specifically, ASFEE can significantly improve kidney function indicators and kidney pathological damage, reverse metabolic disorders caused by DDP, and exert protective effects through multiple pathways such as inhibiting NF-κB p65 activation, regulating the PI3K/Akt pathway to balance Bcl-2/Bax expression, and blocking IL-17 signal transduction. By adopting an integrated strategy that com-bines network pharmacology with experimental verification, the cascading mechanism of ASFEE from “metabolic repair” to “inflammation suppression” and then to “cell protection” was systematically clarified, providing a solid pharmacological basis for the development of ASFEE as a protective drug against chemotherapy-induced kidney injury. Although further exploration is still needed in the identification of specific active ingredients and the validation of gene knockout models, this study provides a new theoretical perspective for understanding the mechanism of action of the metabol-ic-inflammatory network in AKI.

## Data Availability

The original contributions presented in this study are included in the article/Supplementary Materials. Further inquiries can be directed to the corresponding author(s).

## Supplementary Materials

The following supporting information can be downloaded at: https://www.mdpi.com/article/10.3390/ijms262211131/s1.

## Abbreviations

The following abbreviations are used in this manuscript:

ASFEE	*Acanthopanax senticosus* fruit ethanol extract
AKI	acute kidney injury
DDP	cisplatin
UHPLC-Q-TOF/MS	Ultra-High Performance Liquid Chromatography-Quadrupole-Time of Flight/Mass Spectrometry
BUN	Blood Urea Nitrogen
Scr	Serum Creatinine
DEGs	Differentially Expressed Genes
DAMs	Differentially Accumulated Metabolites
ESI	Electrospray Ionization
VIP	Variable Importance in Projection
