# Supplementary material for: Integrative Transcriptomic and Metabolomic Analysis Reveals That Acanthopanax senticosus Fruit Ameliorates Cisplatin-Induced Acute Kidney Injury by Suppressing the NF-κB/PI3K-AKT Pathway via UGT1A1 Regulation
Source: Int J Mol Sci. 2025 Nov 18;26(22):11131. doi: 10.3390/ijms262211131 (PMC12652992; doi:10.3390/ijms262211131)
Supplement: Supplementary file 1 [file ijms-26-11131-s001.zip › ijms-3928310-supplementary.pdf]

Multiomics analysis unveils the protective effects of ethanol extract from acanthopanax senticosus fruit against cisplatin-induced acute kidney injury

Liu Han[1,†], Zebo Tang [2,†], Xiangyu Ma [1,3], Qiuyue Zhang[1], Yu Han[1], Qi Wang[1], Jinlong Liu[1], Xuefeng Bian[1], Liancong Gao[4], Mengran Xu [1,*], Xin Sun [1,*]

[1] College of pharmacy, Jilin Medical University, Jilin132013, China; hanliu@jlmu.cn (L.H.); 1604569375@qq.com (M.X.); zqy45125969@163.com (Q. Z.); hanyu.jlmu@vip.163.com (Y.H.); wangqicvb@163.com (Q.W.); jinlongliu@jlmu.edu.cn; (J.L.); bxfaisy@jlmu.edu.cn (X.B.); sunxinbh@126.com (X.S.)

[2] College of Basic Medical Sciences, Jilin Medical University, Jilin132013, China; tangzebo@jlmu.edu.cn (Z.T.)

[3] College of pharmacy, Yanbian University, Yanji, China; maxy@jlmu.edu.cn (X.M.)

[4] Clinical medical college, Jilin Medical University, Jilin132013, China; gaolc@jlmu.edu.cn (L.G.)

[†] These authors contributed equally to this work.

[*] Correspondence: tangzebo@jlmu.edu.cn (Z.T.) and sunxinbh@126.com (X.S.)

TableS1. Gradient elution conditions of UPLC

Table S2. Identification of chemical components in ASFEE using UPLC-MS/MS

Table S3. Sequencing data statistics

Table S4. Results were compared with reference genomes

Table S5. GO results of all the enriched terms of DEGs (up-regulated by DDP and down-regulated by ASFEE)

Table S6. GO results of all the enriched terms of DEGs (dowm-regulated by DDP and up-regulated by ASFEE)

Table S7. Total ion count and identification statistics

Table S8. Enrichment of KEGG metabolic pathway of DAMs

Table S9. Primer information of genes used for qPCR validation

Figure S1. The TIC diagram of ASFEE samples in the positive ion mode

Figure S2. The TIC diagram of ASFEE samples in negative ion mode

Figure S3. The TIC diagram of control group in kidney tissues in positive ion mode

Figure S4. The TIC diagram of control group in kidney tissues in negative ion mode

Figure S5. The TIC diagram of DDP group in kidney tissues in positive ion mode

Figure S6. The TIC diagram of DDP group in kidney tissues in negative ion mode

Figure S7. The TIC diagram of ASFEE group in kidney tissues in positive ion mode

Figure S8. The TIC diagram of ASFEE group in kidney tissues in negative ion mode

Figure S9. The effects of 1,2,3, 6-tetragalloylglucose (TeGG) on mRNA expression of inflammatory factors and apoptosis-related genes in HK2 cells by RT-qPCR

Figure S10. The effects of PI3K/AKT inhibitor Sophocarpine on the mRNA expression of NFKABIA and RELA genes in HK2 cells by RT-qPCR

Figure S11. Scores (OPLS-DA) plot and Permutation testing in different groups

TableS1. Gradient elution conditions of UPLC

Time	Flow Rate（mL/min）	%A	%B
0	0.3	100	0
10	0.3	70	30
25	0.3	60	40
30	0.3	50	50
40	0.3	30	70
45	0.3	0	100
60	0.3	0	100
60.5	0.3	100	0
70	0.3	100	0

A: Deionized water with 0.1% formic acid; B: Acetonitrile containing 0.1% formic acid

Table S2. Identification of chemical components in ASFEE using UPLC-MS/MS

Name	Molecular formula	Quality deviation（ppm）	Molecular weight	Retention time（min）	Match score	Peak area	Relative content (%)	Key bioactive components
Betaine	$C_5H_{11}NO_2$	1.18	117.07912	1.559	84.6	4385155270	12.684	No
Sucrose	$C_{12}H_{22}O_{11}$	-0.2	342.11614	1.558	94.8	3155094039	9.126	No
Citric acid	$C_6H_8O_7$	0.26	192.02705	1.599	82.6	2877463816	8.323	No
Chlorogenic acid	$C_{16}H_{18}O_9$	0	354.09508	19.079	93.5	1885526766	5.454	Yes
Isoquercitrin	$C_{21}H_{20}O_{12}$	0.71	464.0958	21.56	93	1859114138	5.378	No
3,5-Dicaffeoylquinic acid	$C_{25}H_{24}O_{12}$	0.55	516.12706	22.433	91.9	1376831375	3.983	No
Oleanonic acid	$C_{30}H_{46}O_3$	-0.16	454.34462	27.358	81.7	1155324503	3.342	No
5-Hydroxymethylfurfural	$C_6H_6O_3$	1.35	126.03186	1.52	83.1	1066106331	3.084	No
α-Linolenic acid	$C_{18}H_{30}O_2$	0.83	278.22481	40.03	90	932600125.4	2.698	No

Quinic acid	$C_7H_{12}O_6$	-0.34	192.06332	19.076	91.4	890095805.4	2.575	No
Ursolic acid	$C_{30}H_{48}O_3$	0.47	456.36056	38.086	82	828129857.7	2.395	No
Mannitol	$C_6H_{14}O_6$	-0.33	182.07898	1.487	86.8	602079344.8	1.742	No
Protocatechuic acid	$C_7H_6O_4$	-0.29	154.02656	16.635	87.3	225395505.6	0.652	Yes
Nicotinic acid	$C_6H_5NO_2$	1.81	123.03225	1.566	73.1	209227804.4	0.605	No
Adenosine	$C_{10}H_{13}N_5O_4$	-4.9	267.09545	10.925	78	190653253.1	0.551	No
Uridine	$C_9H_{12}N_2O_6$	1.36	244.06987	5.532	91.4	189300508.4	0.548	No
Eleutheroside E	$C_{34}H_{46}O_{18}$	0.63	742.26888	20.028	88	149268462	0.432	Yes
Rutin	$C_{27}H_{30}O_{16}$	0.64	610.15378	21.132	91.5	133142863.7	0.385	No
Adenine	$C_5H_5N_5$	1.41	135.05469	2.935	77.6	132293101.5	0.383	No
Quercetin	$C_{15}H_{10}O_7$	-0.02	302.04265	25.57	86.8	126263072.8	0.365	Yes
Isofraxidin	$C_{11}H_{10}O_5$	0.6	222.05296	22.05	76.2	115939717.3	0.335	Yes
Cryptochlorogenic acid	$C_{16}H_{18}O_9$	-0.15	354.09503	17.82	87.4	115185118.1	0.333	No
Protocatechualdehyde	$C_7H_6O_3$	-0.69	138.0316	18.177	83.1	96564029.34	0.279	No
Caffeic acid	$C_9H_8O_4$	-0.39	180.04219	19.628	84.2	94407068.06	0.273	Yes
Shikimic acid	$C_7H_{10}O_5$	-0.61	174.05272	2.364	89	92897294.1	0.269	No
Quercitrin	$C_{21}H_{20}O_{11}$	0.66	448.10086	22.499	86.6	91015428.74	0.263	No
Scopoletin	$C_{10}H_8O_4$	1.29	192.04251	19.068	70.6	75090718.86	0.217	No
5,7-Dihydroxychromone	$C_9H_6O_4$	0.03	178.02661	22.688	87.9	71968004.86	0.208	No
Isoalantolactone	$C_{15}H_{20}O_2$	-0.05	232.14632	29.886	73.1	71339240.13	0.206	No
7-Methoxycoumarin	$C_{10}H_8O_3$	1.35	144.02139	20.461	73.1	70338192.44	0.203	No
Astragalin	$C_{21}H_{20}O_{11}$	0.71	448.10088	22.212	88.4	63659893.66	0.184	No
1,3-Dicaffeoylquinic acid	$C_{25}H_{24}O_{12}$	0.23	516.12689	19.912	91.6	56560405.59	0.164	No
1-Caffeoylquinic acid	$C_{16}H_{18}O_9$	-0.19	354.09501	17.289	89.4	52494110.09	0.152	No
Fumaric acid	$C_4H_4O_4$	-1.55	116.01078	4.632	72.4	52225334.87	0.151	Yes

L-Tryptophan	$C_{11}H_{12}N_2O_2$	0.35	204.08995	17.722	85.9	49449676.33	0.143	No
Ferulic acid	$C_{10}H_{10}O_4$	0.71	194.05805	18.967	81	45757676.48	0.132	Yes
Epicatechin	$C_{15}H_{14}O_6$	0.52	290.07919	18.478	85.4	34933314.68	0.101	No
p-Coumaric acid	$C_9H_8O_3$	0.01	164.04735	21.131	82.6	32039063.69	0.093	Yes
Kaempferol	$C_{15}H_{10}O_6$	0.88	286.04799	22.22	81.4	31142925.63	0.090	Yes
Taxifolin	$C_{15}H_{12}O_7$	-0.1	304.05827	22.039	92.1	30851902.16	0.089	Yes
Quercetin 3-O-β-D-Glucuronide	$C_{21}H_{18}O_{13}$	0.33	478.0749	21.627	88.3	26220768.91	0.076	Yes
Coumarin	$C_9H_6O_2$	0.39	146.03684	20.048	71	23268753.59	0.067	No
Salicylic acid	$C_7H_6O_3$	-0.69	138.0316	23.327	80.2	23025408.02	0.067	No
Isorhamnetin	$C_1\ H_{12}O_7$	0.69	316.05852	22.514	85.1	22630412.09	0.065	No
Benzoic acid	$C_7H_6O_2$	1.34	122.03694	18.702	81.2	14157308.89	0.041	No

Table S3. Sequencing data statistics

Sample	Raw reads	Raw bases	Clean reads	Clean bases	Error rate (%)	Q20(%)	Q30(%)	GC content (%)
Control_1	44454552	6712637352	44170388	6619268501	0.0117	98.97	96.6	45.85
Control_2	49626416	7493588816	49323380	7400823500	0.0116	99.01	96.74	46.25
Control_3	44865904	6774751504	44588584	6686084033	0.0117	98.97	96.57	46.06
Control_4	50771090	7666434590	50466556	7566982577	0.0116	98.99	96.64	45.69
Control_5	40177214	6066759314	39913388	5990930374	0.0117	98.97	96.61	46.55
Control_6	47013730	7099073230	46700244	7006084063	0.0116	99.03	96.79	46.23
Model_1	50627652	7644775452	50258720	7543960762	0.0116	98.98	96.64	46.75

Model_2	48944610	7390636110	48606036	7294346973	0.0116	99.01	96.75	46.84
Model_3	42006456	6342974856	41692240	6256420147	0.0116	98.97	96.64	46.87
Model_4	55624260	8399263260	55198384	8274079227	0.0117	98.97	96.62	46.99
Model_5	42080006	6354080906	41784280	6268063462	0.0117	98.97	96.59	46.46
Model_6	48029122	7252397422	47693408	7147374733	0.0116	98.98	96.66	47
ASFEE_1	42144906	6363880806	41862064	6279712773	0.0117	98.97	96.59	46
ASFEE_2	46397496	7006021896	46086420	6905410417	0.0116	99.01	96.73	46.61
ASFEE_3	49941420	7541154420	49598616	7440457192	0.0117	98.95	96.49	46.31
ASFEE_4	49506264	7475445864	49154588	7375696250	0.0116	98.99	96.67	46.19
ASFEE_5	51164004	7725764604	50819482	7617609506	0.0117	98.96	96.55	46.59
ASFEE_6	42079814	6354051914	41799666	6273244017	0.0117	98.96	96.55	45.83

Table S4. Results were compared with reference genomes

Sample	Total reads	Total mapped	Multiple mapped	Unique mapped
Control_1	44170388	43559331(98.62%)	6141865(13.9%)	37417466(84.71%)
Control_2	49323380	48634109(98.6%)	6624590(13.43%)	42009519(85.17%)
Control_3	44588584	43962768(98.6%)	5981621(13.42%)	37981147(85.18%)
Control_4	50466556	49775029(98.63%)	7426126(14.71%)	42348903(83.91%)
Control_5	39913388	39340827(98.57%)	5236076(13.12%)	34104751(85.45%)
Control_6	46700244	46037265(98.58%)	6515499(13.95%)	39521766(84.63%)
Model_1	50258720	49428099(98.35%)	6097274(12.13%)	43330825(86.22%)
Model_2	48606036	47788017(98.32%)	5686160(11.7%)	42101857(86.62%)
Model_3	41692240	40963413(98.25%)	5021487(12.04%)	35941926(86.21%)
Model_4	55198384	54250973(98.28%)	6712060(12.16%)	47538913(86.12%)
Model_5	41784280	41061014(98.27%)	5524076(13.22%)	35536938(85.05%)
Model_6	47693408	46873973(98.28%)	5656895(11.86%)	41217078(86.42%)
ASFEE_1	41862064	41254736(98.55%)	5784678(13.82%)	35470058(84.73%)
ASFEE_2	46086420	45426405(98.57%)	5845882(12.68%)	39580523(85.88%)
ASFEE_3	49598616	48878451(98.55%)	6524986(13.16%)	42353465(85.39%)

ASFEE_4	49154588	48441670(98.55%)	6691353(13.61%)	41750317(84.94%)
ASFEE_5	50819482	50086727(98.56%)	6576086(12.94%)	43510641(85.62%)
ASFEE_6	41799666	41175229(98.51%)	5996051(14.34%)	35179178(84.16%)

Table S5. GO results of all the enriched terms of DEGs (up-regulated by DDP and down-regulated by ASFEE)

Important1: the intersection genes between the downregulated genes in the ASFEE vs. model comparison and the upregulated genes in the model vs. control comparison

GO id	Term description	Term type	Important 1 number	Important 1 percent
GO:0001906	cell killing	biological_process	15	15/ 2201
GO:0002376	immune system process	biological_process	269	269/ 2201
GO:0065007	biological regulation	biological_process	1097	1097/ 2201
GO:0008152	metabolic process	biological_process	577	577/ 2201
GO:0098743	cell aggregation	biological_process	3	3/ 2201
GO:0043473	pigmentation	biological_process	4	4/ 2201
GO:0051704	multi-organism process	biological_process	212	212/ 2201
GO:0040011	locomotion	biological_process	136	136/ 2201
GO:0022414	reproductive process	biological_process	134	134/ 2201
GO:0000003	reproduction	biological_process	2	2/ 2201
GO:0008283	cell population proliferation	biological_process	62	62/ 2201
GO:0071840	cellular component organization or biogenesis	biological_process	427	427/ 2201
GO:0009987	cellular process	biological_process	1204	1204/ 2201
GO:0032502	developmental process	biological_process	545	545/ 2201
GO:0032501	multicellular organismal process	biological_process	306	306/ 2201
GO:0040007	growth	biological_process	33	33/ 2201
GO:0048511	rhythmic process	biological_process	26	26/ 2201
GO:0051179	localization	biological_process	314	314/ 2201
GO:0022610	biological adhesion	biological_process	124	124/ 2201
GO:0007610	behavior	biological_process	58	58/ 2201

GO:0098754	detoxification	biological_process	7	7/ 2201
GO:0023052	signaling	biological_process	58	58/ 2201
GO:0050896	response to stimulus	biological_process	624	624/ 2201
GO:0031974	membrane-enclosed lumen	cellular_component	39	39/ 2201
GO:0032991	protein-containing complex	cellular_component	395	395/ 2201
GO:0044456	synapse part	cellular_component	96	96/ 2201
GO:0005623	cell	cellular_component	14	14/ 2201
GO:0044425	membrane part	cellular_component	642	642/ 2201
GO:0044421	extracellular region part	cellular_component	326	326/ 2201
GO:0044422	organelle part	cellular_component	644	644/ 2201
GO:0043226	organelle	cellular_component	833	833/ 2201
GO:0045202	synapse	cellular_component	73	73/ 2201
GO:0016020	membrane	cellular_component	682	682/ 2201
GO:0030054	cell junction	cellular_component	105	105/ 2201
GO:0005576	extracellular region	cellular_component	124	124/ 2201
GO:0009295	nucleoid	cellular_component	1	1/ 2201
GO:0044217	other organism part	cellular_component	5	5/ 2201
GO:0044464	cell part	cellular_component	1407	1407/ 2201
GO:0099080	supramolecular complex	cellular_component	76	76/ 2201
GO:0045182	translation regulator activity	molecular_function	9	9/ 2201
GO:0140110	transcription regulator activity	molecular_function	107	107/ 2201
GO:0005198	structural molecule activity	molecular_function	89	89/ 2201
GO:0044183	protein folding chaperone	molecular_function	1	1/ 2201
GO:0038024	cargo receptor activity	molecular_function	15	15/ 2201
GO:0016209	antioxidant activity	molecular_function	11	11/ 2201
GO:0005215	transporter activity	molecular_function	87	87/ 2201
GO:0098772	molecular function regulator	molecular_function	250	250/ 2201
GO:0005488	binding	molecular_function	1236	1236/ 2201

GO:0031386	protein tag	molecular_function	1	1/ 2201
GO:0060089	molecular transducer activity	molecular_function	127	127/ 2201
GO:0003824	catalytic activity	molecular_function	465	465/ 2201

Table S6. GO results of all the enriched terms of DEGs (dowm-regulated by DDP and up-regulated by ASFEE)

Important 2: the intersection genes between the upregulated genes in the ASFEE vs. model comparison and the downregulated genes in the model vs. control comparison

GO id	Term description	Term type	Important 1 number	Important 1 percent
GO:0001906	cell killing	biological_process	2	2/ 1573
GO:0002376	immune system process	biological_process	52	52/ 1573
GO:0065007	biological regulation	biological_process	638	638/ 1573
GO:0008152	metabolic process	biological_process	528	528/ 1573
GO:0043473	pigmentation	biological_process	3	3/ 1573
GO:0051704	multi-organism process	biological_process	58	58/ 1573
GO:0040011	locomotion	biological_process	51	51/ 1573
GO:0022414	reproductive process	biological_process	81	81/ 1573
GO:0000003	reproduction	biological_process	2	2/ 1573
GO:0008283	cell population proliferation	biological_process	24	24/ 1573
GO:0071840	cellular component organization or biogenesis	biological_process	255	255/ 1573
GO:0009987	cellular process	biological_process	863	863/ 1573
GO:0032502	developmental process	biological_process	328	328/ 1573
GO:0032501	multicellular organismal process	biological_process	215	215/ 1573
GO:0040007	growth	biological_process	27	27/ 1573
GO:0048511	rhythmic process	biological_process	22	22/ 1573
GO:0051179	localization	biological_process	285	285/ 1573
GO:0022610	biological adhesion	biological_process	33	33/ 1573
GO:0009758	carbohydrate utilization	biological_process	1	1/ 1573

GO:0007610	behavior	biological_process	45	45/ 1573
GO:0098754	detoxification	biological_process	6	6/ 1573
GO:0023052	signaling	biological_process	18	18/ 1573
GO:0019740	nitrogen utilization	biological_process	1	1/ 1573
GO:0050896	response to stimulus	biological_process	307	307/ 1573
GO:0031974	membrane-enclosed lumen	cellular_component	69	69/ 1573
GO:0032991	protein-containing complex	cellular_component	233	233/ 1573
GO:0044456	synapse part	cellular_component	88	88/ 1573
GO:0005623	cell	cellular_component	4	4/ 1573
GO:0044425	membrane part	cellular_component	546	546/ 1573
GO:0044421	extracellular region part	cellular_component	131	131/ 1573
GO:0044422	organelle part	cellular_component	489	489/ 1573
GO:0043226	organelle	cellular_component	647	647/ 1573
GO:0045202	synapse	cellular_component	77	77/ 1573
GO:0016020	membrane	cellular_component	516	516/ 1573
GO:0030054	cell junction	cellular_component	75	75/ 1573
GO:0005576	extracellular region	cellular_component	70	70/ 1573
GO:0009295	nucleoid	cellular_component	2	2/ 1573
GO:0044217	other organism part	cellular_component	1	1/ 1573
GO:0044464	cell part	cellular_component	1030	1030/ 1573
GO:0099080	supramolecular complex	cellular_component	32	32/ 1573
GO:0045182	translation regulator activity	molecular_function	7	7/ 1573
GO:0140110	transcription regulator activity	molecular_function	42	42/ 1573
GO:0005198	structural molecule activity	molecular_function	22	22/ 1573
GO:0044183	protein folding chaperone	molecular_function	2	2/ 1573
GO:0038024	cargo receptor activity	molecular_function	8	8/ 1573
GO:0016209	antioxidant activity	molecular_function	9	9/ 1573
GO:0005215	transporter activity	molecular_function	148	148/ 1573

GO:0098772	molecular function regulator	molecular_function	90	90/ 1573
GO:0140299	small molecule sensor activity	molecular_function	2	2/ 1573
GO:0005488	binding	molecular_function	818	818/ 1573
GO:0060089	molecular transducer activity	molecular_function	79	79/ 1573
GO:0003824	catalytic activity	molecular_function	583	583/ 1573

Table S7. Total ion count and identification statistics

Ion mode	All peaks(raw)	Identified metabolites(raw)	Effective peaks(origin)	Identified metabolites(origin)
pos	2684	759	1989	583
neg	4409	1180	3438	980

Table S8. Enrichment of KEGG metabolic pathway of DAMs

Num	First Category	Second Category	Pathway Description	Pathway_ID	Enrich Factor	Ratio_in_pop	P_value	P_adjust
1	Metabolism	Carbohydrate metabolism	Fructose and mannose metabolism	map00051	0.017857143	56/4518	0.5467	0.6184
1	Metabolism	Carbohydrate metabolism	Galactose metabolism	map00052	0.02173913	46/4518	0.4775	0.5884
1	Metabolism	Nucleotide metabolism	Purine metabolism	map00230	0.00990099	101/4518	0.7617	0.7729
1	Metabolism	Nucleotide metabolism	Pyrimidine metabolism	map00240	0.015625	64/4518	0.5955	0.6522
1	Metabolism	Amino acid metabolism	Alanine, aspartate and glutamate metabolism	map00250	0.035714286	28/4518	0.3259	0.4888
1	Metabolism	Amino acid metabolism	Valine, leucine and isoleucine biosynthesis	map00290	0.043478261	23/4518	0.2766	0.4241
1	Metabolism	Amino acid metabolism	Tyrosine metabolism	map00350	0.012820513	78/4518	0.6687	0.721
1	Metabolism	Amino acid metabolism	Phenylalanine, tyrosine and tryptophan biosynthesis	map00400	0.028571429	35/4518	0.3894	0.5269

1	Metabolism	Metabolism of other amino acids	Phosphonate and phosphinate metabolism	map00440	0.017857143	56/4518	0.5467	0.6184
1	Metabolism	Metabolism of other amino acids	Glutathione metabolism	map00480	0.026315789	38/4518	0.4148	0.54
1	Metabolism	Biosynthesis of other secondary metabolites	Neomycin, kanamycin and gentamicin biosynthesis	map00524	0.012345679	81/4518	0.6826	0.7137
1	Metabolism	Glycan biosynthesis and metabolism	Glycosylphosphatidylinositol (GPI)-anchor biosynthesis	map00563	0.142857143	7/4518	0.09368	0.3402
1	Metabolism	Lipid metabolism	Arachidonic acid metabolism	map00590	0.012658228	79/4518	0.6734	0.7149
1	Metabolism	Lipid metabolism	alpha-Linolenic acid metabolism	map00592	0.022727273	44/4518	0.4625	0.5802
1	Metabolism	Lipid metabolism	Sphingolipid metabolism	map00600	0.028571429	35/4518	0.3894	0.5269
1	Metabolism	Carbohydrate metabolism	Pyruvate metabolism	map00620	0.03125	32/4518	0.363	0.5111
1	Metabolism	Carbohydrate metabolism	Butanoate metabolism	map00650	0.021276596	47/4518	0.4849	0.587
1	Metabolism	Metabolism of cofactors and vitamins	Thiamine metabolism	map00730	0.032258065	31/4518	0.3539	0.5087
1	Metabolism	Metabolism of cofactors and vitamins	Biotin metabolism	map00780	0.034482759	29/4518	0.3354	0.4923
1	Metabolism	Xenobiotics biodegradation and metabolism	Drug metabolism - cytochrome P450	map00982	0.011494253	87/4518	0.7087	0.7299
1	Metabolism	Global and overview maps	Biosynthesis of nucleotide sugars	map01250	0.004975124	201/4518	0.9443	0.9443
1	Environmental Information Processing	Signal transduction	Sphingolipid signaling pathway	map04071	0.066666667	15/4518	0.1902	0.375

1	Environmental Information Processing	Signal transduction	AMPK signaling pathway	map04152	0.045454545	22/4518	0.2663	0.4375
1	Cellular Processes	Cell growth and death	Apoptosis	map04210	0.25	4/4518	0.05464	0.3427
1	Cellular Processes	Cell growth and death	Necroptosis	map04217	0.1	10/4518	0.1311	0.377
1	Organismal Systems	Nervous system	Serotonergic synapse	map04726	0.023809524	42/4518	0.4471	0.5712
1	Organismal Systems	Sensory system	Taste transduction	map04742	0.03125	32/4518	0.363	0.5111
1	Organismal Systems	Endocrine system	Insulin secretion	map04911	0.083333333	12/4518	0.1553	0.3571
1	Organismal Systems	Endocrine system	Regulation of lipolysis in adipocytes	map04923	0.071428571	14/4518	0.1787	0.3737
1	Organismal Systems	Endocrine system	Aldosterone synthesis and secretion	map04925	0.045454545	22/4518	0.2663	0.4375
1	Human Diseases	Endocrine and metabolic disease	Type II diabetes mellitus	map04930	0.166666667	6/4518	0.08085	0.3719
1	Human Diseases	Endocrine and metabolic disease	Insulin resistance	map04931	0.052631579	19/4518	0.2346	0.426
1	Organismal Systems	Digestive system	Protein digestion and absorption	map04974	0.021276596	47/4518	0.4849	0.587
1	Organismal Systems	Digestive system	Cholesterol metabolism	map04979	0.1	10/4518	0.1311	0.377
1	Human Diseases	Neurodegenerative disease	Prion disease	map05020	0.2	5/4518	0.06783	0.36
2	Metabolism	Carbohydrate metabolism	Glycolysis / Gluconeogenesis	map00010	0.0625	32/4518	0.07266	0.3581
2	Metabolism	Carbohydrate metabolism	Citrate cycle (TCA cycle)	map00020	0.1	20/4518	0.03094	0.3559
2	Metabolism	Carbohydrate metabolism	Pentose phosphate pathway	map00030	0.054054054	37/4518	0.09338	0.379
2	Metabolism	Lipid metabolism	Primary bile acid biosynthesis	map00120	0.042553191	47/4518	0.139	0.3552

2	Metabolism	Amino acid metabolism	Glycine, serine and threonine metabolism	map00260	0.041666667	48/4518	0.1438	0.3543
2	Metabolism	Amino acid metabolism	Cysteine and methionine metabolism	map00270	0.029850746	67/4518	0.2397	0.424
2	Metabolism	Amino acid metabolism	Lysine degradation	map00310	0.035714286	56/4518	0.1833	0.3719
2	Metabolism	Amino acid metabolism	Arginine and proline metabolism	map00330	0.028985507	69/4518	0.2501	0.4314
2	Metabolism	Amino acid metabolism	Phenylalanine metabolism	map00360	0.040816327	49/4518	0.1486	0.3536
2	Metabolism	Metabolism of other amino acids	Taurine and hypotaurine metabolism	map00430	0.083333333	24/4518	0.04338	0.4276
2	Metabolism	Metabolism of other amino acids	D-Amino acid metabolism	map00470	0.028985507	69/4518	0.2501	0.4314
2	Metabolism	Carbohydrate metabolism	Amino sugar and nucleotide sugar metabolism	map00520	0.016806723	119/4518	0.4982	0.5827
2	Metabolism	Carbohydrate metabolism	Glyoxylate and dicarboxylate metabolism	map00630	0.03125	64/4518	0.2241	0.418
2	Metabolism	Metabolism of cofactors and vitamins	Riboflavin metabolism	map00740	0.083333333	24/4518	0.04338	0.4276
2	Metabolism	Metabolism of cofactors and vitamins	Nicotinate and nicotinamide metabolism	map00760	0.036363636	55/4518	0.1782	0.3843
2	Metabolism	Metabolism of cofactors and vitamins	Pantothenate and CoA biosynthesis	map00770	0.066666667	30/4518	0.06485	0.3729
2	Metabolism	Metabolism of cofactors and vitamins	Lipoic acid metabolism	map00785	0.045454545	44/4518	0.1248	0.3915
2	Metabolism	Metabolism of terpenoids and polyketides	Terpenoid backbone biosynthesis	map00900	0.043478261	46/4518	0.1342	0.3562

2	Metabolism	Xenobiotics biodegradation and metabolism	Metabolism of xenobiotics by cytochrome P450	map00980	0.016528926	121/4518	0.5072	0.5832
2	Metabolism	Lipid metabolism	Biosynthesis of unsaturated fatty acids	map01040	0.027027027	74/4518	0.2761	0.433
2	Metabolism	Global and overview maps	Nucleotide metabolism	map01232	0.034482759	58/4518	0.1934	0.3706
2	Environmental Information Processing	Signal transduction	HIF-1 signaling pathway	map04066	0.133333333	15/4518	0.01788	0.3084
2	Organismal Systems	Endocrine system	Glucagon signaling pathway	map04922	0.076923077	26/4518	0.05019	0.3463
2	Organismal Systems	Digestive system	Vitamin digestion and absorption	map04977	0.051282051	39/4518	0.1021	0.3523
2	Human Diseases	Cancer: overview	Central carbon metabolism in cancer	map05230	0.054054054	37/4518	0.09338	0.379
2	Human Diseases	Cardiovascular disease	Diabetic cardiomyopathy	map05415	0.051282051	39/4518	0.1021	0.3523
3	Metabolism	Lipid metabolism	Steroid hormone biosynthesis	map00140	0.03030303	99/4518	0.159	0.354
3	Metabolism	Lipid metabolism	Linoleic acid metabolism	map00591	0.107142857	28/4518	0.006603	0.2278
4	Metabolism	Carbohydrate metabolism	Pentose and glucuronate interconversions	map00040	0.051724138	58/4518	0.04635	0.3553
4	Metabolism	Amino acid metabolism	Tryptophan metabolism	map00380	0.048192771	83/4518	0.02778	0.3834
4	Environmental Information Processing	Membrane transport	ABC transporters	map02010	0.028985507	138/4518	0.1252	0.3756
5	Metabolism	Carbohydrate metabolism	Ascorbate and aldarate metabolism	map00053	0.087719298	57/4518	0.001076	0.07426
6	Organismal Systems	Digestive system	Bile secretion	map04976	0.051546392	97/4518	0.01082	0.2488

9	Metabolism	Global and overview maps	Biosynthesis of cofactors	map01240	0.024316109	329/4518	0.08461	0.3649

Table S9. Primer information of genes used for qPCR validation

Gene	Sequence (5' – 3')	Forward/Reverse
Rela	AGGCTTCTGGGCCTTATGTG	F
	TGCTTCTCTCGCCAGGAATAC	R
Trp53	CTCTCCCCCGCAAAAGAAAAA	F
	CGGAACATCTCGAAGCGTTTA	R

Nfkbia	TGAAGGACGAGGAGTACGAGC	F
	TTCGTGGATGATTGCCAAGTG	R
Cdkn1a	CCTGGTGATGTCCGACCTG	F
	CCATGAGCGCATCGCAATC	R
Mdm2	TGTCTGTGTCTACCGAGGGTG	F
	TCCAACGGACTTTAACAACTTCA	R
Pik3r5	TGCTCTGGAGCGATGCTTG	F
	ACCTCTTGGGTCTTTTGTAGGA	R
Rras2	TGTGACGGACTATGATCCAACC	F
	ATTGCTCTCTCATGGCTCCAA	R
Nras	ACTGAGTACAAACTGGTGGTGG	F
	TCGGTAAGAATCCTCTATGGTGG	R
Myc	CCCTATTTCATCTGCGACGAG	F
	GAGAAGGACGTAGCGACCG	R
IL6	CTGCAAGAGACTTCCATCCAG	F
	AGTGGTATAGACAGGTCTGTTGG	R
IL1B	GCAACTGTTCCTGAACTCAACT	F
	ATCTTTTGGGGTCCGTCAACT	R
TNF	CCTGTAGCCCACGTCGTAG	F
	GGGAGTAGACAAGGTACAACCC	R
Ugt1a1	ACAGGGAGCTAGAGTCTGGG	F
	CCCCCAAAGAGCCATGTGAT	R
Ugt1a9	ACAGGGAGCTAGAGTCTGGG	F
	CCCCCAAAGAGCCATGTGAT	R
Ugt2a3	CCGGTTGTCATGAGTGAGCT	F

	GGGCCTTCCTAGGGTTTCAC	R
Ugt2b37	TATGGTGGCCAAAGGAGCAG	F
	AGGCTGGTCATGGTGAATGG	R
Ugt2b38	GAGTGTACAAGTGGCTCCCC	F
	CTGCTCCTTTGGCCACCATA	R
Ugt1a7c	ACAGGGAGCTAGAGTCTGGG	F
	CCCCCAAAGAGCCATGTGAT	R

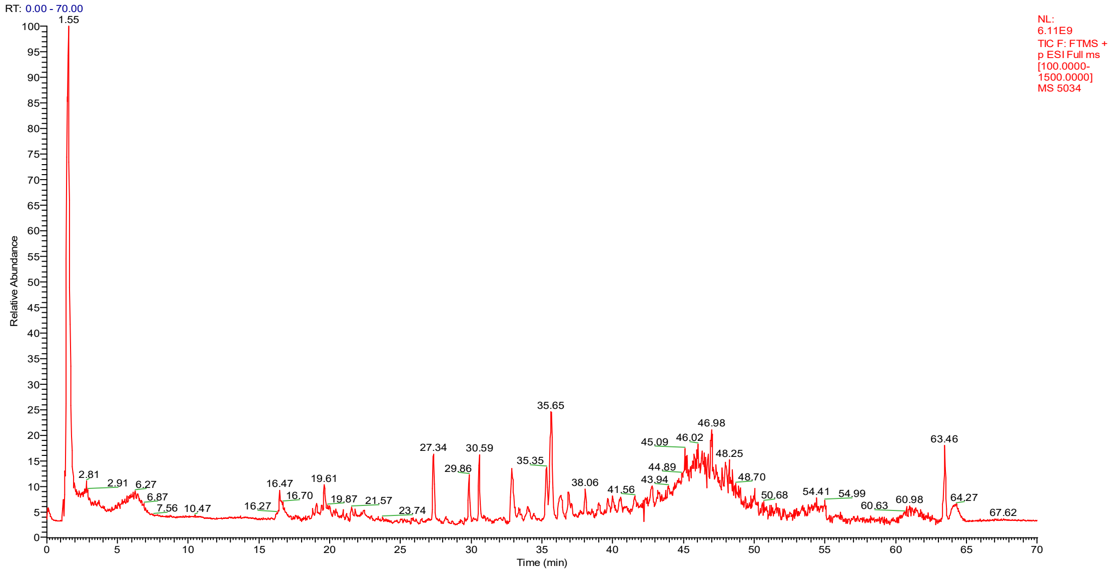

Figure S1. The TIC diagram of ASFEE samples in the positive ion mode

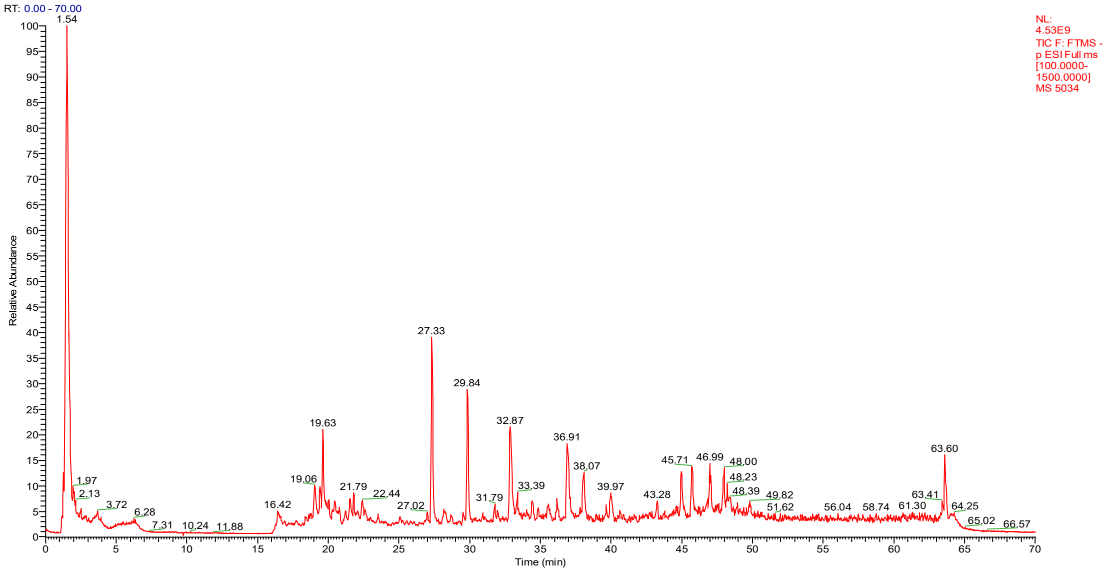

Figure S2. The TIC diagram of ASFEE samples in negative ion mode

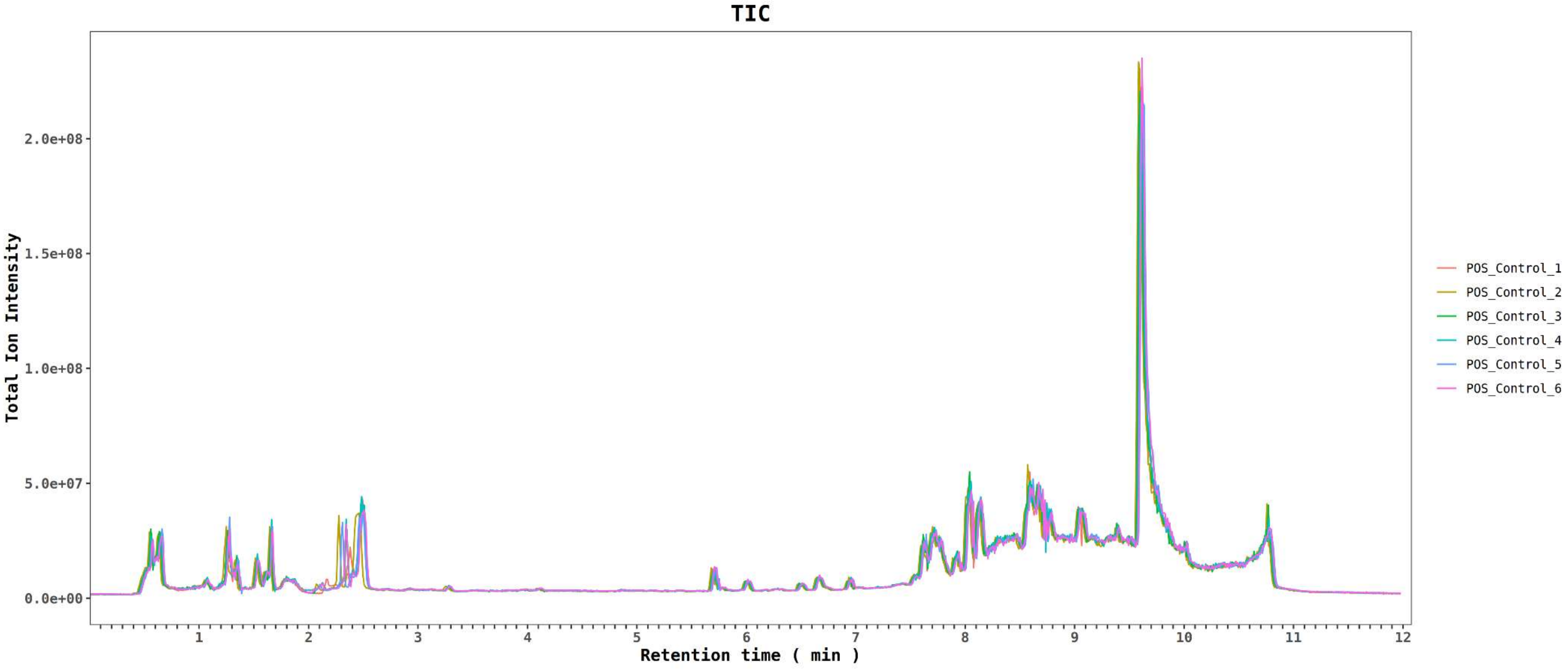

Figure S3. The TIC diagram of control group in kidney tissues in positive ion mode.

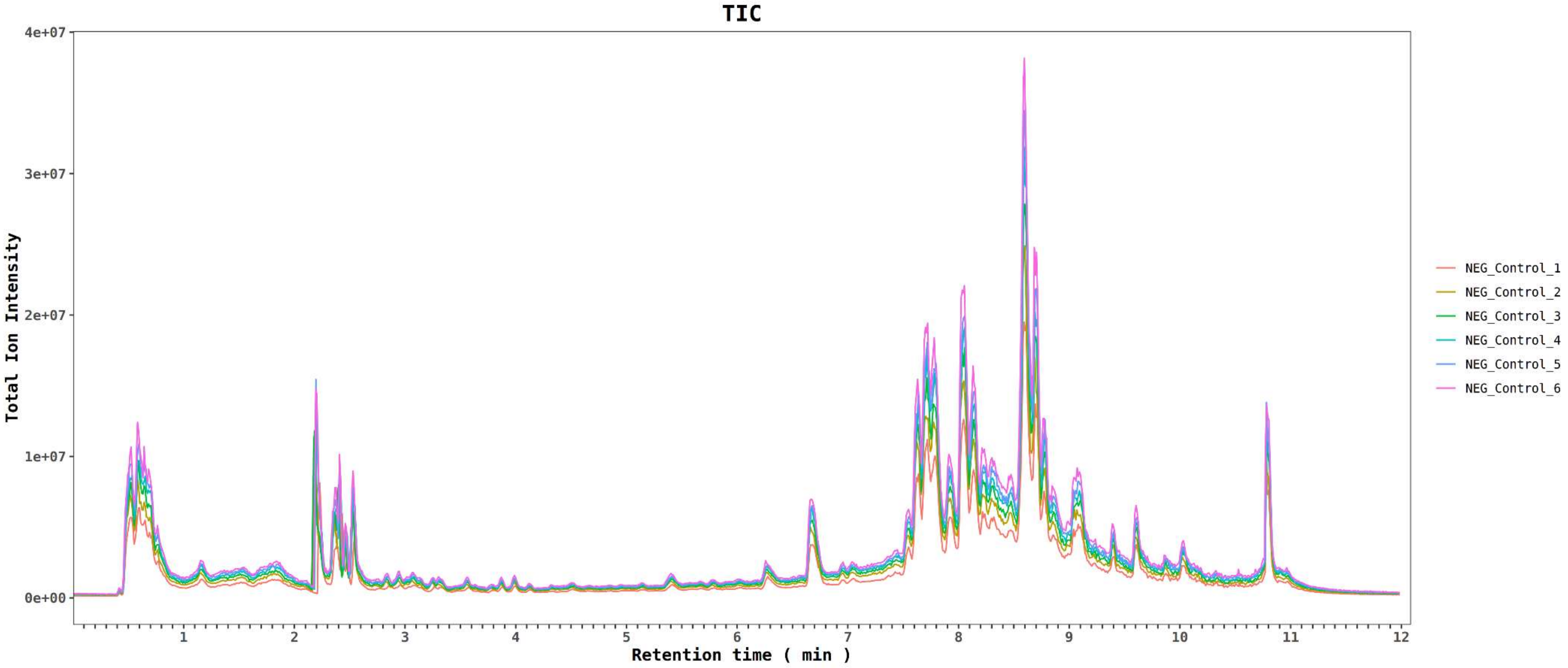

Figure S4. The TIC diagram of control group in kidney tissues in negative ion mode.

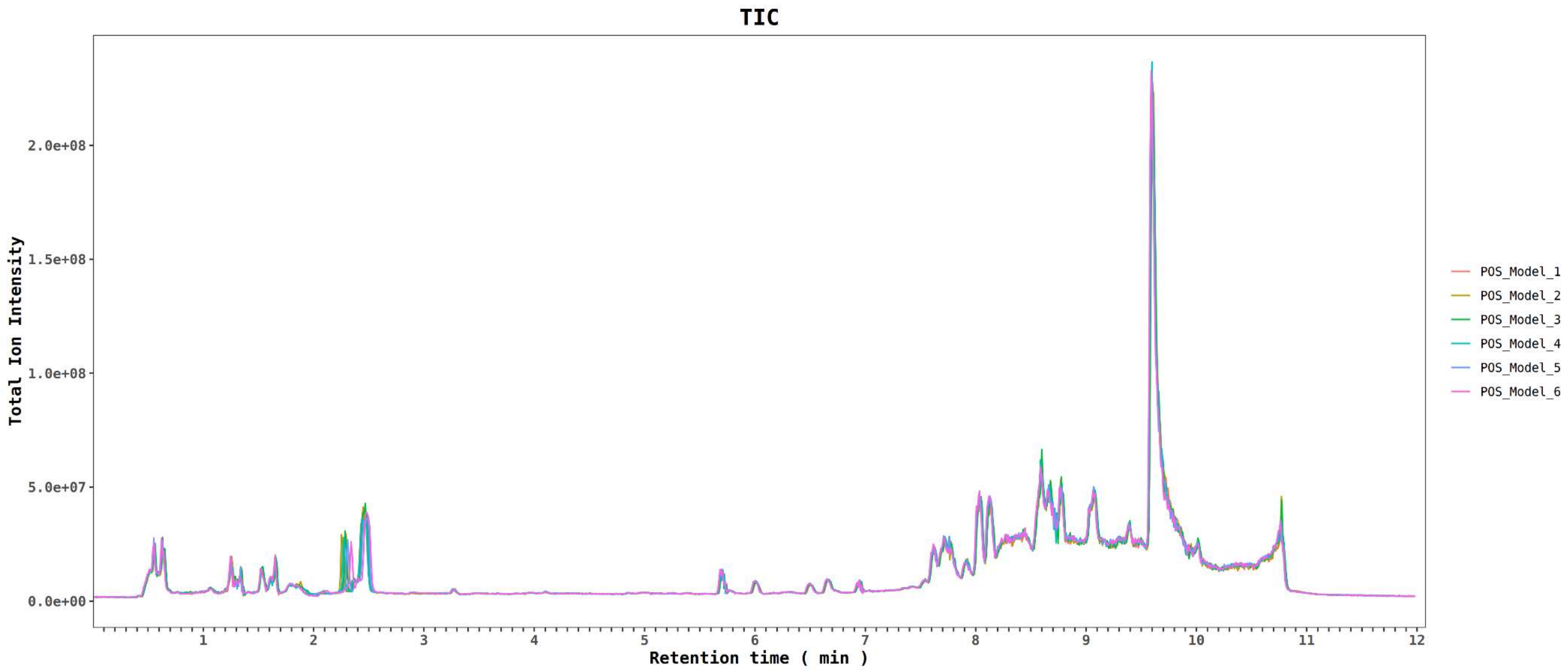

Figure S5. The TIC diagram of DDP group in kidney tissues in positive ion mode.

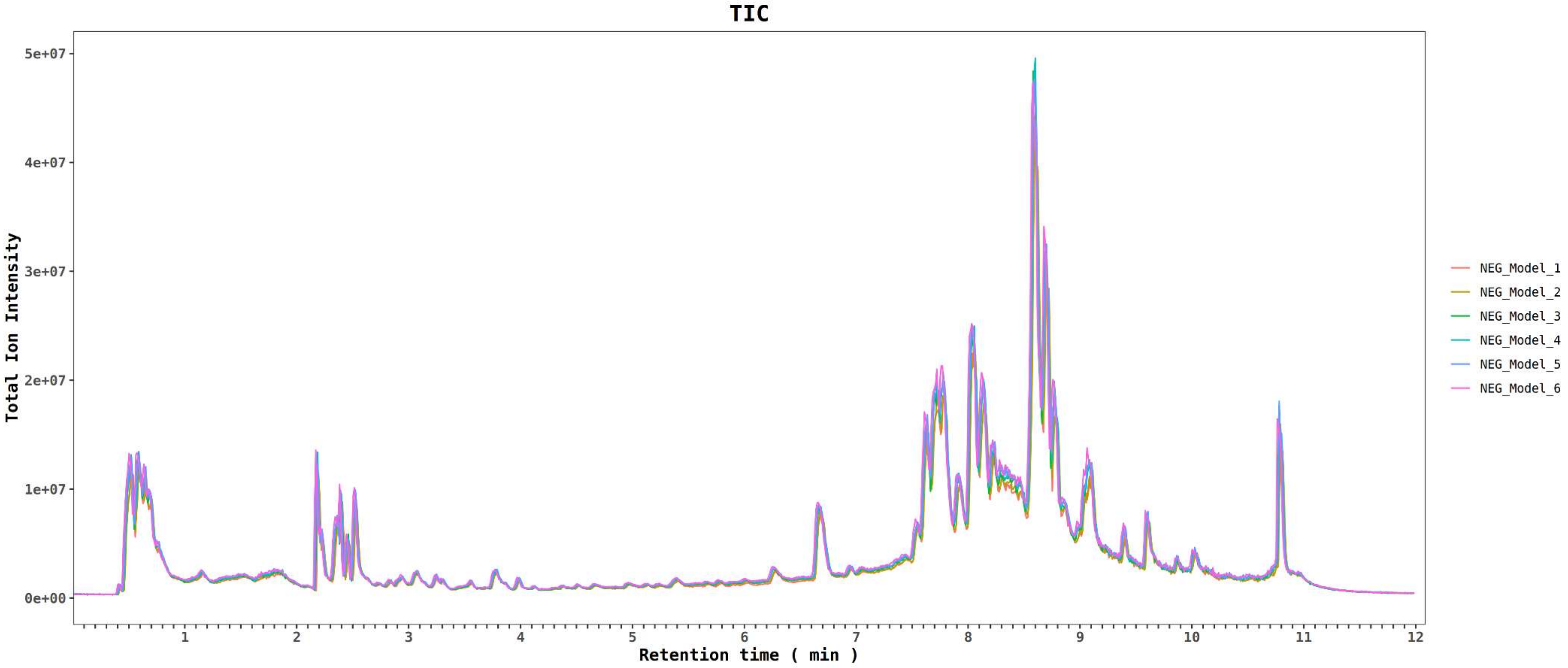

Figure S6. The TIC diagram of DDP group in kidney tissues in negative ion mode.

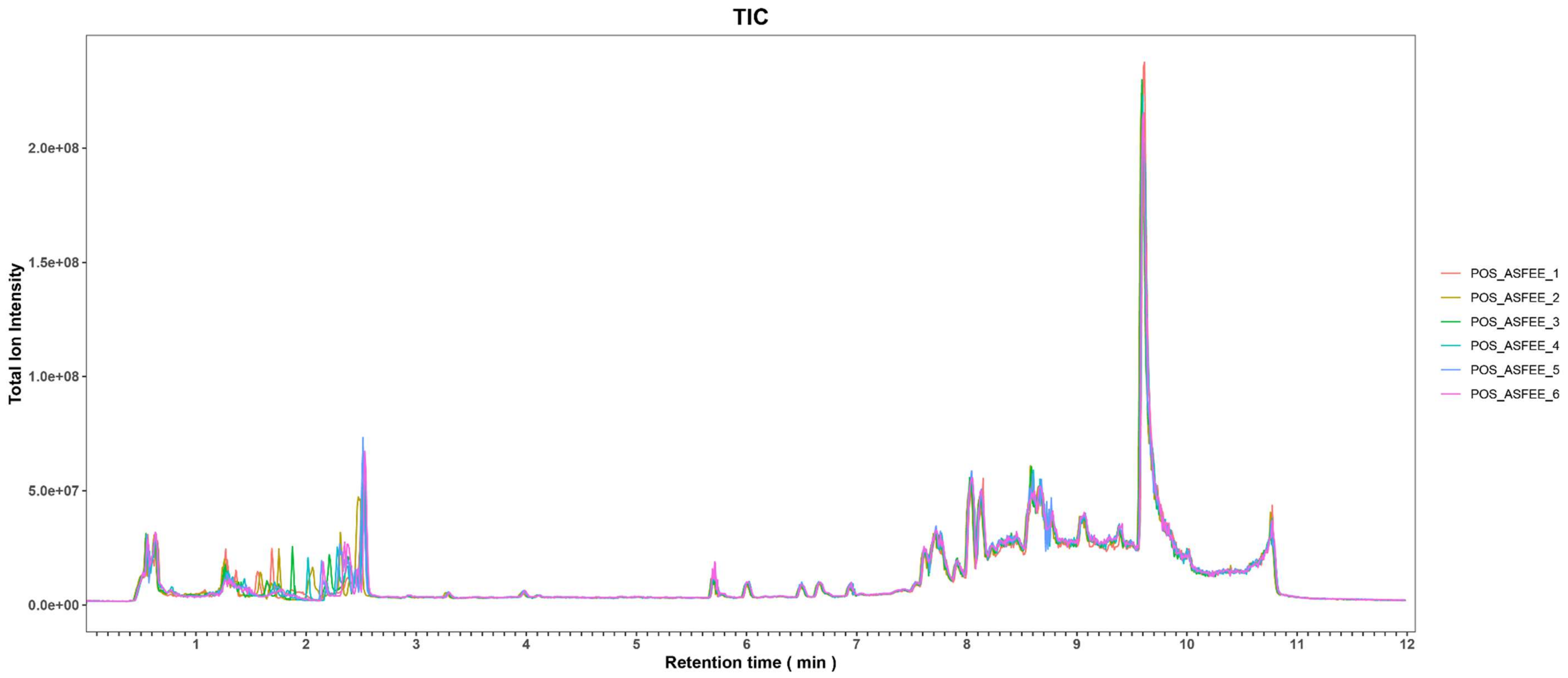

Figure S7. The TIC diagram of ASFEE group in kidney tissues in positive ion mode.

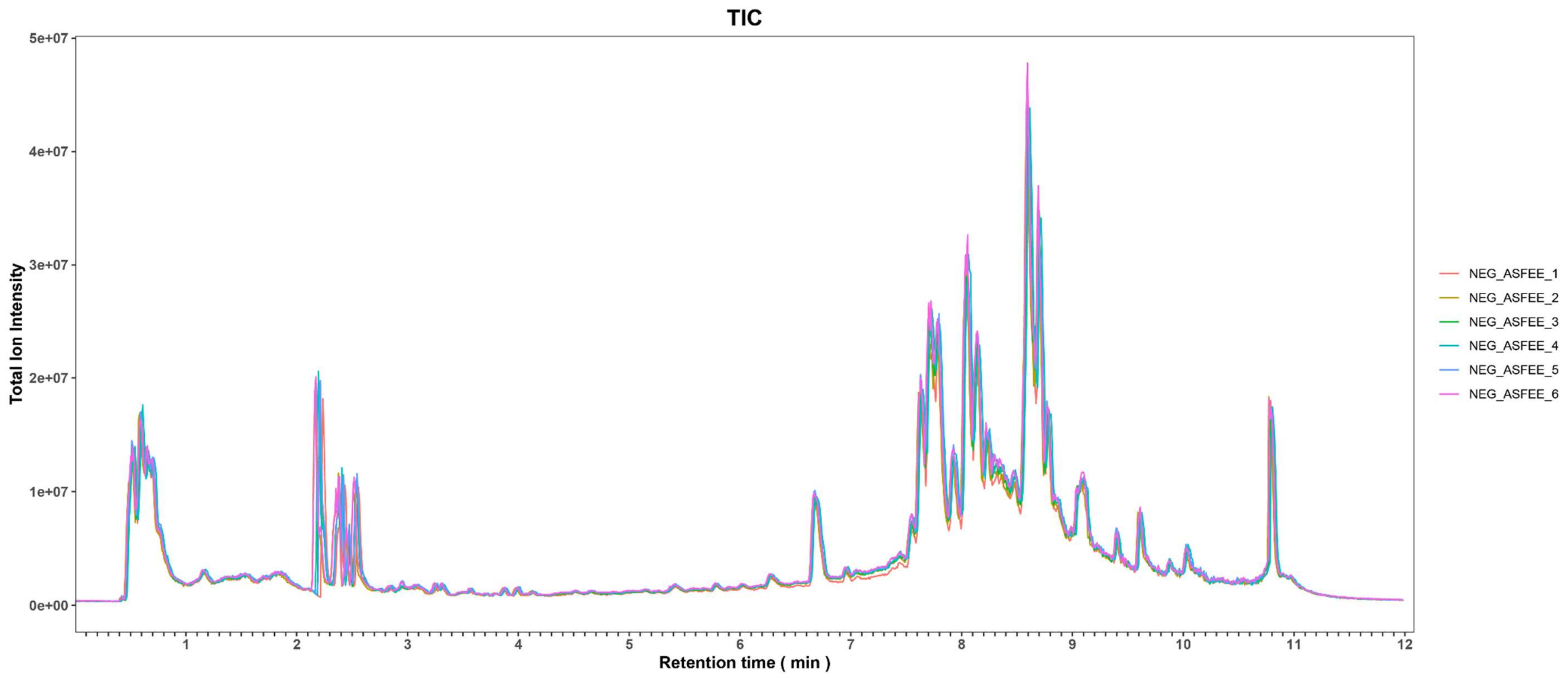

Figure S8. The TIC diagram of ASFEE group in kidney tissues in negative ion mode.

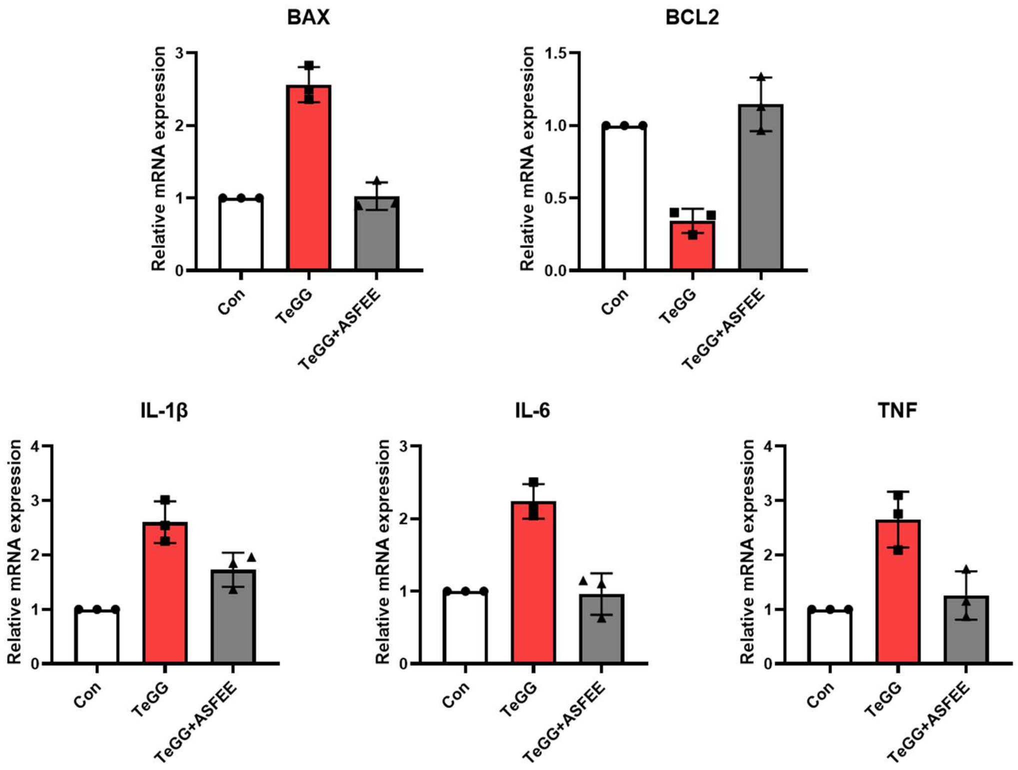

Figure S9. The effects of 1,2,3, 6-tetragalloylglucose (TeGG) on mRNA expression of inflammatory factors and apoptosis-related genes in HK2 cells by RT-qPCR

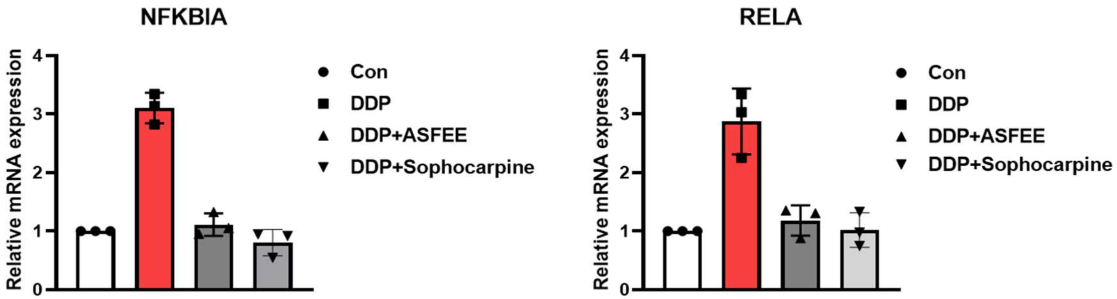

Figure S10. The effects of PI3K/AKT inhibitor Sophocarpine on the mRNA expression of NFKABIA and RELA genes in HK2 cells by RT-qPCR

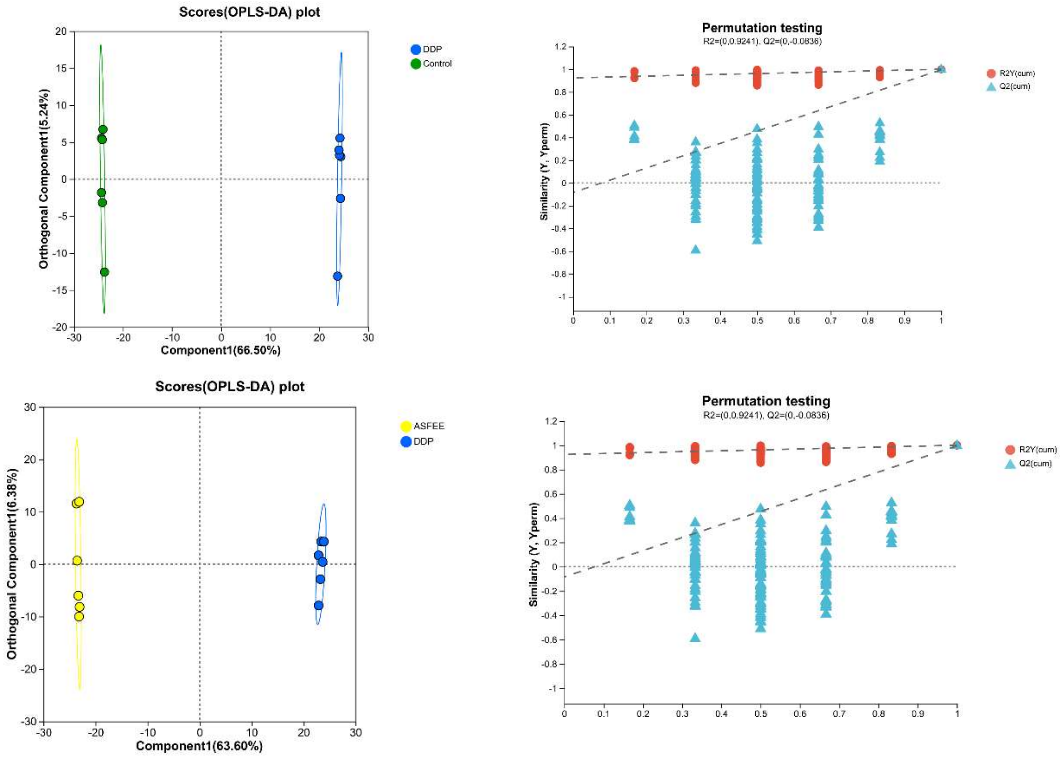

Figure S11. Scores (OPLS-DA) plot and Permutation testing in different groups
